# Reverse-transcribed SARS-CoV-2 RNA can integrate into the genome of cultured human cells and can be expressed in patient-derived tissues

**DOI:** 10.1073/pnas.2105968118

**Published:** 2021-05-06

**Authors:** Liguo Zhang, Alexsia Richards, M. Inmaculada Barrasa, Stephen H. Hughes, Richard A. Young, Rudolf Jaenisch

**Affiliations:** ^a^Whitehead Institute for Biomedical Research, Cambridge, MA 02142;; ^b^HIV Dynamics and Replication Program, Center for Cancer Research, National Cancer Institute, Frederick, MD 21702;; ^c^Department of Biology, Massachusetts Institute of Technology, Cambridge, MA 02142

**Keywords:** SARS-CoV-2, reverse transcription, LINE1, genomic integration, chimeric RNAs

## Abstract

Prolonged detection of severe acute respiratory syndrome coronavirus 2 (SARS-CoV-2) RNA and recurrence of PCR-positive tests have been widely reported in patients after recovery from COVID-19, but some of these patients do not appear to shed infectious virus. We investigated the possibility that SARS-CoV-2 RNAs can be reverse-transcribed and integrated into the DNA of human cells in culture and that transcription of the integrated sequences might account for some of the positive PCR tests seen in patients. In support of this hypothesis, we found that DNA copies of SARS-CoV-2 sequences can be integrated into the genome of infected human cells. We found target site duplications flanking the viral sequences and consensus LINE1 endonuclease recognition sequences at the integration sites, consistent with a LINE1 retrotransposon-mediated, target-primed reverse transcription and retroposition mechanism. We also found, in some patient-derived tissues, evidence suggesting that a large fraction of the viral sequences is transcribed from integrated DNA copies of viral sequences, generating viral–host chimeric transcripts. The integration and transcription of viral sequences may thus contribute to the detection of viral RNA by PCR in patients after infection and clinical recovery. Because we have detected only subgenomic sequences derived mainly from the 3′ end of the viral genome integrated into the DNA of the host cell, infectious virus cannot be produced from the integrated subgenomic SARS-CoV-2 sequences.

## Significance

An unresolved issue of SARS-CoV-2 disease is that patients often remain positive for viral RNA as detected by PCR many weeks after the initial infection in the absence of evidence for viral replication. We show here that SARS-CoV-2 RNA can be reverse-transcribed and integrated into the genome of the infected cell and be expressed as chimeric transcripts fusing viral with cellular sequences. Importantly, such chimeric transcripts are detected in patient-derived tissues. Our data suggest that, in some patient tissues, the majority of all viral transcripts are derived from integrated sequences. Our data provide an insight into the consequence of SARS-CoV-2 infections that may help to explain why patients can continue to produce viral RNA after recovery.

Continuous or recurrent positive severe acute respiratory syndrome coronavirus 2 (SARS-CoV-2) PCR tests have been reported in samples taken from patients weeks or months after recovery from an initial infection (1–17). Although bona fide reinfection with SARS-CoV-2 after recovery has recently been reported (18), cohort-based studies with subjects held in strict quarantine after they recovered from COVID-19 suggested that at least some “re-positive” cases were not caused by reinfection (19, 20). Furthermore, no replication-competent virus was isolated or spread from these PCR-positive patients (1–3, 5, 6, 12, 16), and the cause for the prolonged and recurrent production of viral RNA remains unknown. SARS-CoV-2 is a positive-stranded RNA virus. Like other beta-coronaviruses (SARS-CoV-1 and Middle East respiratory syndrome-related coronavirus), SARS-CoV-2 employs an RNA-dependent RNA polymerase to replicate its genomic RNA and transcribe subgenomic RNAs (21–24). One possible explanation for the continued detection of SARS-CoV-2 viral RNA in the absence of virus reproduction is that, in some cases, DNA copies of viral subgenomic RNAs may integrate into the DNA of the host cell by a reverse transcription mechanism. Transcription of the integrated DNA copies could be responsible for positive PCR tests long after the initial infection was cleared. Indeed, nonretroviral RNA virus sequences have been detected in the genomes of many vertebrate species (25, 26), with several integrations exhibiting signals consistent with the integration of DNA copies of viral mRNAs into the germline via ancient long interspersed nuclear element (LINE) retrotransposons (reviewed in ref. 27). Furthermore, nonretroviral RNA viruses such as vesicular stomatitis virus or lymphocytic choriomeningitis virus (LCMV) can be reverse transcribed into DNA copies by an endogenous reverse transcriptase (RT), and DNA copies of the viral sequences have been shown to integrate into the DNA of host cells (28–30). In addition, cellular RNAs, for example the human *APP* transcripts, have been shown to be reverse-transcribed by endogenous RT in neurons with the resultant APP fragments integrated into the genome and expressed (31). Human LINE1 elements (∼17% of the human genome), a type of autonomous retrotransposons, which are able to retro-transpose themselves and other nonautonomous elements such as Alu, are a source of cellular endogenous RT (32–34). Endogenous LINE1 elements have been shown to be expressed in aged human tissues (35) and LINE1-mediated somatic retrotransposition is common in cancer patients (36, 37). Moreover, expression of endogenous LINE1 and other retrotransposons in host cells is commonly up-regulated upon viral infection, including SARS-CoV-2 infection (38–40).

In this study, we show that SARS-CoV-2 sequences can integrate into the host cell genome by a LINE1-mediated retroposition mechanism. We provide evidence that the integrated viral sequences can be transcribed and that, in some patient samples, the majority of viral transcripts appear to be derived from integrated viral sequences.

## Results

### Integration of SARS-CoV-2 Sequences into the DNA of Host Cells in Culture.

We used three different approaches to detect genomic SARS-CoV-2 sequences integrated into the genome of infected cells. These approaches were Nanopore long-read sequencing, Illumina paired-end whole genomic sequencing, and Tn5 tagmentation-based DNA integration site enrichment sequencing. All three methods provided evidence that SARS-CoV-2 sequences can be integrated into the genome of the host cell.

To increase the likelihood of detecting rare integration events, we transfected HEK293T cells with LINE1 expression plasmids prior to infection with SARS-CoV-2 and isolated DNA from the cells 2 d after infection (*SI Appendix*, Fig. S1*A*). We detected DNA copies of SARS-CoV-2 nucleocapsid (NC) sequences in the infected cells by PCR (*SI Appendix*, Fig. S1*B*) and cloned the complete NC gene (*SI Appendix*, Fig. S1*D*) from large-fragment cell genomic DNA that had been gel-purified (*SI Appendix*, Fig. S1*C*). The viral DNA sequence (NC) was confirmed by Sanger sequencing (Dataset S1). These results suggest that SARS-CoV-2 RNA can be reverse-transcribed, and the resulting DNA could be integrated into the genome of the host cell.

**Fig. 1.**
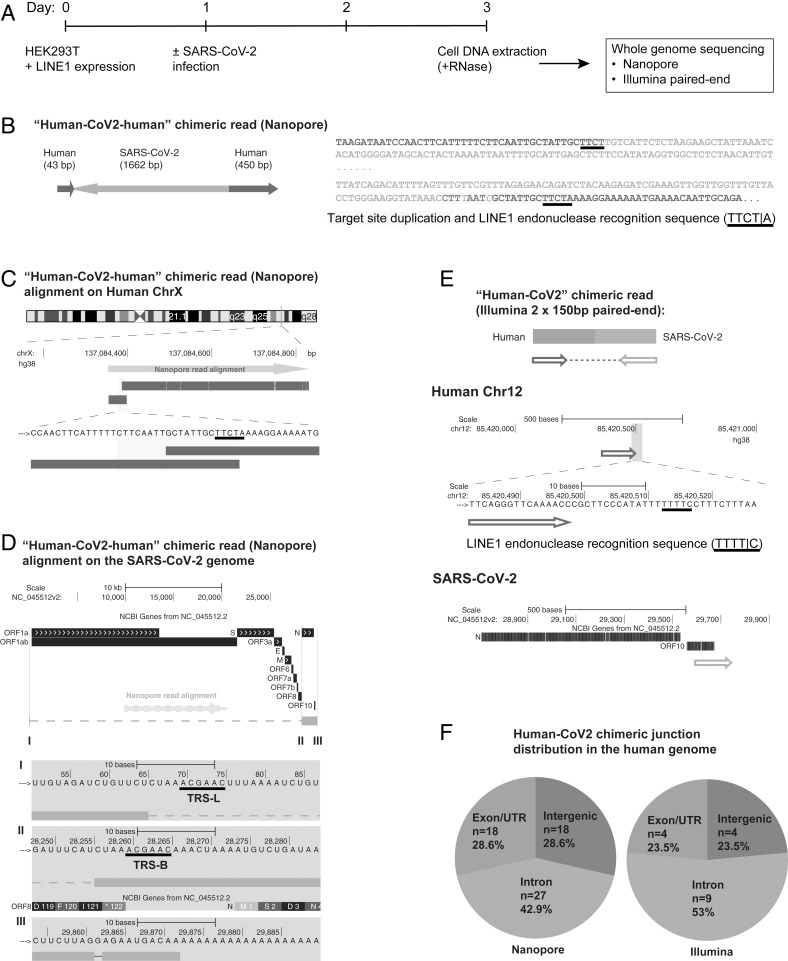
SARS-CoV-2 RNA can be reverse transcribed and integrated into the host cell genome. (*A*) Experimental workflow. (*B*) Chimeric sequence from a Nanopore sequencing read showing integration of a full-length SARS-CoV-2 NC subgenomic RNA sequence (magenta) and human genomic sequences (blue) flanking both sides of the integrated viral sequence. Features indicative of LINE1-mediated “target-primed reverse transcription” include the target site duplication (yellow highlight) and the LINE1 endonuclease recognition sequence (underlined). Sequences that could be mapped to both genomes are shown in purple with mismatches to the human genomic sequences in italics. The arrows indicate sequence orientation with regard to the human and SARS-CoV-2 genomes as shown in *C* and *D*. (*C*) Alignment of the Nanopore read in *B* with the human genome (chromosome X) showing the integration site. The human sequences at the junction region show the target site, which was duplicated when the SARS-CoV-2 cDNA was integrated (yellow highlight) and the LINE1 endonuclease recognition sequence (underlined). (*D*) Alignment of the Nanopore read in *B* with the SARS-CoV-2 genome showing the integrated viral DNA is a copy of the full-length NC subgenomic RNA. The light blue highlighted regions are enlarged to show TRS-L (I) and TRS-B (II) sequences (underlined, these are the sequences where the viral polymerase jumps to generate the subgenomic RNA) and the end of the viral sequence at the poly(A) tail (III). These viral sequence features (I–III) show that a DNA copy of the full-length NC subgenomic RNA was retro-integrated. (*E*) A human–viral chimeric read pair from Illumina paired-end whole-genome sequencing. The read pair is shown with alignment to the human (blue) and SARS-CoV-2 (magenta) genomes. The arrows indicate the read orientations relative to the human and SARS-CoV-2 genomes. The highlighted (light blue) region of the human read mapping is enlarged to show the LINE1 recognition sequence (underlined). (*F*) Distributions of human–CoV2 chimeric junctions from Nanopore (*Left*) and Illumina (*Right*) sequencing with regard to features of the human genome.

To demonstrate directly that the SARS-CoV-2 sequences were integrated into the host cell genome, DNA isolated from infected LINE1-overexpressing HEK293T cells was used for Nanopore long-read sequencing (Fig. 1*A*). Fig. 1 *B*–*D* shows an example of a full-length viral NC subgenomic RNA sequence (1,662 bp) integrated into the cell chromosome X and flanked on both sides by host DNA sequences. Importantly, the flanking sequences included a 20-bp direct repeat. This target site duplication is a signature of LINE1-mediated retro-integration (41, 42). Another viral integrant comprising a partial NC subgenomic RNA sequence that was flanked by a duplicated host cell DNA target sequence is shown in *SI Appendix*, Fig. S2 *A*–*C*. In both cases, the flanking sequences contained a consensus recognition sequence of the LINE1 endonuclease (43). These results indicate that SARS-CoV-2 sequences can be integrated into the genomes of cultured human cells by a LINE1-mediated retroposition mechanism. Table 1 summarizes all of the linked SARS-CoV-2–host sequences that were recovered. DNA copies of portions of the viral genome were found in almost all human chromosomes. In addition to the two examples given in Fig. 1 and *SI Appendix*, Fig. S2, we also recovered cellular sequences for 61 integrants for which only one of the two host–viral junctions was retrieved (*SI Appendix*, Fig. S2 *D*–*F* and Table 1; Nanopore reads containing the chimeric sequences summarized in Dataset S2). Importantly, about 67% of the flanking human sequences included either a consensus or a variant LINE1 endonuclease recognition sequence (such as TTTT/A) (*SI Appendix*, Fig. S2 *D*–*F* and Table 1). These LINE1 recognition sequences were either at the chimeric junctions that were directly linked to the 3′ end (poly-A tail) of viral sequences, or within a distance of 8–27 bp from the junctions that were linked to the 5′ end of viral sequences, which is within the potential target site duplication. Both results are consistent with a model in which LINE1-mediated retroposition provides a mechanism to integrate DNA copies of SARS-CoV-2 subgenomic fragments into host genomic DNA. About 71% of the viral sequences were flanked by intron or intergenic cellular sequences and 29% by exons (Fig. 1*F* and Table 1). Thus, the association of the viral sequences with exons is much higher than would be expected for random integration into the genome [human genome: 1.1% exons, 24% introns, and 75% intergenic DNA (44)], suggestive of preferential integration into exon-associated target sites. While previous studies showed no preference for LINE1 retroposition into exons (45, 46), our finding suggests that LINE1-mediated retroposition of some other RNAs may be different. We noted that viral–cellular boundaries were frequently close to the 5′ or 3′ untranslated regions (UTRs) of the cellular genes, suggesting that there is a preference for integration close to promoters or poly(A) sites in our experimental system.

**Table 1.** Summary of the human-CoV2 chimeric sequences obtained by Nanopore DNA sequencing of infected LINE1-overexpressing HEK293T cells

	Number of sequences with human-CoV2 junction	With LINE1 recognition sequence at/near junction (e.g., TTTT/A)	Junction at human intergenic	Junction at human intron	Junction at human exon/UTR
chr1	10	6	0	6	4
chr2	2	2	0	2	0
chr3	3	3	0	3	0
chr4	2	2	0	1	1
chr5	1	1	0	1	0
chr6	4	2	3	0	1
chr7	2	2	1	1	0
chr8	0	0	0	0	0
chr9	4	2	0	2	2
chr10	5	1	2	1	2
chr11	3	2	1	1	1
chr12	6	4	2	2	2
chr13	3	3	3	0	0
chr14	2	2	1	1	0
chr15	0	0	0	0	0
chr16	2	1	1	1	0
chr17	2	0	1	0	1
chr18	2	1	0	2	0
chr19	1	1	0	0	1
chr20	0	0	0	0	0
chr21	2	1	1	1	0
chr22	1	1	0	1	0
chrX	6	5	2	1	3
Total	63	42	18	27	18
Fraction		66.7%	28.6%	42.9%	28.6%

To confirm the integration of SARS-CoV-2 sequences into genomic DNA by another method, we subjected DNA isolated from LINE1-transfected and SARS-CoV-2–infected HEK293T cells to Illumina paired-end whole-genome sequencing, using a Tn5-based library construction method (Illumina Nextera) to avoid ligation artifacts. Viral DNA reads were concentrated at the 3′ end of the SARS-CoV-2 genome (*SI Appendix*, Fig. S3). We recovered 17 viral integrants (sum of two replicates), by mapping human–viral chimeric DNA sequences (Fig. 1*E* and Table 2, chimeric sequences summarized in Dataset S3); 7 (41%) of the junctions contained either a consensus or a variant LINE1 recognition sequence in the cellular sequences near the junction (Fig. 1*E* and Table 2), consistent with a LINE1-mediated retroposition mechanism. Similar to the results obtained from Nanopore sequencing, about 76% of the viral sequences were flanked by intron or intergenic cellular sequences and 24% by exons (Fig. 1*F* and Table 2).

**Table 2.** Summary of the human-CoV2 chimeric sequences obtained by Illumina paired-end whole-genome DNA sequencing of infected LINE1-overexpressing HEK293T cells

Region features (human)	Intergenic	Intron	Exon/UTR
Region number	4	9	4
With L1 recognition sequence at/near junction	2	3	2

About 32% of SARS-CoV-2 sequences (6/21 integration events in Nanopore, 4/10 in Illumina data) were integrated at LINEs, short interspersed nuclear elements, or long terminal repeat elements without evidence for a LINE1 recognition site, suggesting that there may be an alternative reverse transcription/integration mechanism, possibly similar to that reported for cells acutely infected with LCMV, which resulted in integrated LCMV sequences fused to intracisternal A-type particle (IAP) sequences (29).

**Fig. 2.**
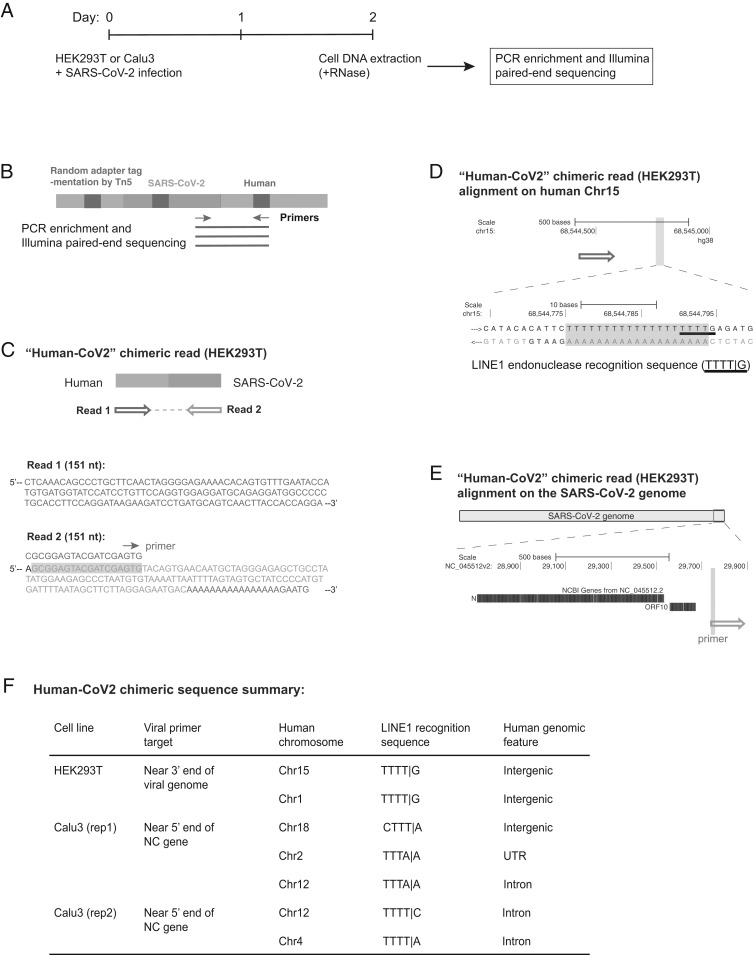
Evidence for integration of SARS-CoV-2 cDNA in cultured cells that do not overexpress a reverse transcriptase. (*A*) Experimental workflow. (*B*) Experimental design for the Tn5 tagmentation-mediated enrichment sequencing method used to map integration sites in the host cell genome. (*C*) A human–viral chimeric read pair supporting viral integration. The reads are aligned with the human (blue) and SARS-CoV-2 (magenta) genomic sequences. The arrows indicate the read orientations relative to the human and SARS-CoV-2 genomes as shown in *D* and *E*. Sequence of the viral primer used for enrichment is shown with green highlight in the read (corresponding to the green arrow illustrated in *B*). Sequences that could be mapped to both genomes are shown in purple. (*D*) Alignment of the read pair in *C* with the human genome (chromosome 15, blue arrow). The highlighted (light blue) region of the human sequence is enlarged to show the LINE1 recognition sequence (underlined) with a 19-base poly-dT sequence (purple highlight) that could be annealed by the viral poly-A tail for “target-primed reverse transcription.” Additional 5-bp human sequence (GAATG, blue) was captured in read 2 (*C*), supporting a bona fide integration site. (*E*) Alignment of the read pair in *C* with the SARS-CoV-2 genome (magenta). The viral primer sequence is shown with green highlight. (*F*) Summary of seven human–viral chimeric sequences identified by the enrichment sequencing method in the two cell lines showing the integrated human chromosomes, LINE1 recognition sequences close to the chimeric junction, and human genomic features at the read junction.

To assess whether genomic integration of SARS-CoV-2 sequences could also occur in infected cells that did not overexpress RT, we isolated DNA from virus-infected HEK293T and Calu3 cells that were not transfected with an RT expression plasmid (Fig. 2*A*). Tn5 tagmentation-mediated DNA integration site enrichment sequencing (47, 48) (Fig. 2*B* and *SI Appendix*, Fig. S4*A*) detected a total of seven SARS-CoV-2 sequences fused to cellular sequences in these cells (sum of three independent infections of two cell lines), all of which showed LINE1 recognition sequences close to the human–SARS-CoV-2 sequence junctions (Fig. 2 *C*–*F* and *SI Appendix*, Fig. S4 *B*–*D*, chimeric sequences summarized in Dataset S4).

### Expression of Viral–Cellular Chimeric Transcripts in Infected Cultured Cells and Patient-Derived Tissues.

To investigate the possibility that SARS-CoV-2 sequences integrated into the genome can be expressed, we analyzed published RNA-seq data from SARS-CoV-2–infected cells for evidence of chimeric transcripts (49). Examination of these datasets (50–55) (*SI Appendix*, Fig. S5) revealed a number of human–viral chimeric reads (*SI Appendix*, Fig. S6 *A* and *B*). These occurred in multiple sample types, including cultured cells and organoids from lung/heart/brain/stomach tissues (*SI Appendix*, Fig. S6*B*). The abundance of the chimeric reads positively correlated with viral RNA level across the sample types (*SI Appendix*, Fig. S6*B*). Chimeric reads generally accounted for 0.004–0.14% of the total SARS-CoV-2 reads in the samples. A majority of the chimeric junctions mapped to the sequence of the SARS-CoV-2 NC gene (*SI Appendix*, Fig. S6 *C* and *D*). This is consistent with the finding that NC RNA is the most abundant SARS-CoV-2 subgenomic RNA (56), making it the most likely target for reverse transcription and integration. However, recent data showed that up to 1% of RNA-seq reads from SARS-CoV-2–infected cells can be artifactually chimeric as a result of RT switching between RNA templates, which can occur during the cDNA synthesis step in the preparation of a RNA-seq library (57). Thus, because there is a mixture of host mRNAs and positive-strand viral mRNAs in infected cells, the identification of genuine chimeric viral–cellular RNA transcripts is compromised by the generation of artifactual chimeras in the assays.

**Fig. 3.**
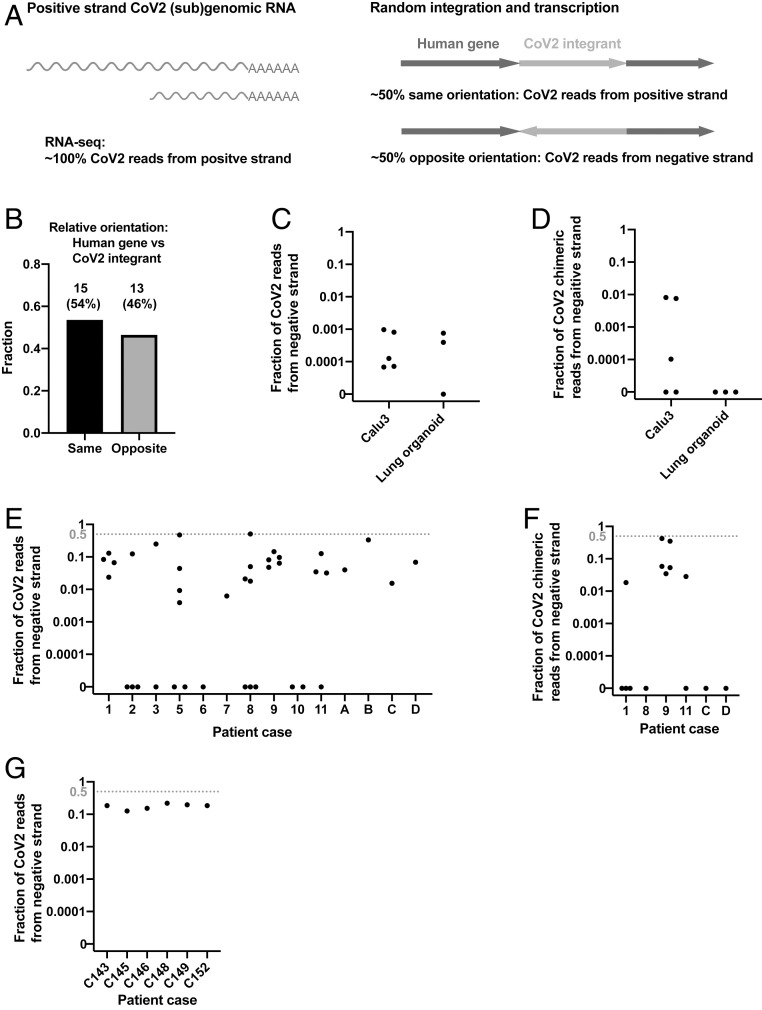
Negative-strand viral RNA-seq reads suggest that integrated SARS-CoV-2 sequences are expressed. (*A*) Schema predicting fractions of positive- or negative-strand SARS-CoV-2 RNA-seq reads that are derived from viral (sub)genomic RNAs or from transcripts of integrated viral sequences. The arrows (*Right*) showing the orientation of an integrated SARS-CoV-2 (magenta) positive strand relative to the orientation of the host cellular gene (blue). (*B*) Fractions of SARS-CoV-2 sequences integrated into human genes with same (*n* = 15) or opposite (*n* = 13) orientation of the viral positive strand relative to the positive strand of the human gene. A total of 28 integration events at human genes with LINE1 endonuclease recognition sequences were identified from our Nanopore DNA sequencing of infected LINE1-overexpressing HEK293T cells (Fig. 1*A*). (*C*) Fraction of total viral reads that are derived from negative-strand viral RNA in acutely infected cells or organoids (see *SI Appendix*, Table S1 for details). (*D*) Fraction of human–viral chimeric reads that contain viral sequences derived from negative-strand viral RNA in acutely infected cells or organoids (see *SI Appendix*, Table S1 for details). (*E*) Fraction of total viral reads that are derived from negative-strand viral RNA in published patient RNA-seq data (autopsy FFPE samples, GSE150316, samples with no viral reads or of low library strandedness quality not included; see *SI Appendix*, Table S2 for details; reanalysis results consistent with the original publication). (*F*) Fraction of human–viral chimeric reads that contain viral sequences derived from negative-strand viral RNA in published patient RNA-seq data (autopsy FFPE samples, GSE150316; see *SI Appendix*, Table S2 for details). (*G*) Fraction of total viral reads that are derived from negative-strand viral RNA in published patient RNA-seq data (BALF samples, GSE145926; see *SI Appendix*, Table S3 for details). The red dashed lines in *E*–*G* indicate the level at which 50% of all viral reads (*E* and *G*) or viral sequences in human–viral chimeric reads (*F*) were from negative-strand viral RNAs, a level expected if all the viral sequences were derived from integrated sequences.

We reasoned that the orientation of an integrated DNA copy of SARS-CoV-2 RNA should be random with respect to the orientation of the targeted host gene, predicting that about half the viral DNAs that were integrated into an expressed host gene should be in an orientation opposite to the direction of the host cell gene’s transcription (Fig. 3*A*). As predicted, ∼50% of viral integrants in human genes were in the opposite orientation relative to the host gene in our Nanopore dataset (integration at human genes with LINE1 recognition sequences, Fig. 3*B*). Thus, for chimeric transcripts derived from integrated viral sequences, we would expect that ∼50% of the chimeric transcripts should contain negative-strand viral sequences linked to positive-strand host RNA sequences. We therefore determined the fraction of the viral and human–viral chimeric transcripts in infected cultured cells/organoids and in patient-derived tissues containing negative-strand viral RNA sequences.

The replication of SARS-CoV2 RNA requires the synthesis of negative-strand viral RNA, which serves as template for replication of viral genomic RNA and transcription of viral subgenomic positive-strand RNA (21). To assess the prevalence of negative-strand viral RNA in acutely infected cells, we determined the ratio of total positive to negative-strand RNAs. Between 0 and 0.1% of total viral reads were derived from negative-strand RNA in acutely infected Calu3 cells or lung organoids [our data and published data (50, 58)] (Fig. 3*C* and *SI Appendix*, Table S1), similar to what has been reported in clinical samples taken early after infection (59). These results argue that the level of negative-strand viral RNA is at least 1,000-fold lower than that of positive-strand viral RNA in acutely infected cells, due at least in part to a massive production of positive-strand subgenomic RNA during viral replication. This greatly reduces the likelihood that random template switching during the reverse transcription step in the RNA-seq library construction would generate a large fraction of the artifactual chimeric reads that would contain viral negative-strand RNA fused to cellular positive-strand RNA sequences. We determined that between 0 and 1% of human–viral chimeric reads contained negative-strand viral sequences in the acutely infected cells/organoids (Fig. 3*D* and *SI Appendix*, Table S1), consistent with a small fraction of viral reads being derived from integrated SARS-CoV-2 sequences.

In contrast to the results obtained with acutely infected Calu3 cells or lung organoids, up to 51% of all viral reads, and up to 42.5% of human–viral chimeric reads, were derived from the negative-strand SARS-CoV-2 RNA in some patient-derived tissues [published data (60, 61), patient clinical background available in the original publications] (Fig. 3 *E*–*G* and *SI Appendix*, Tables S2 and S3). Single-cell analysis of patient lung bronchoalveolar lavage fluid (BALF) cells from patients with severe COVID [published data (61)] showed that up to 40% of all viral reads were derived from the negative-strand SARS-CoV-2 RNA (*SI Appendix*, Fig. S7). Fractions of negative-strand RNA in tissues from some patients were orders of magnitude higher than those in acutely infected cells or organoids (Fig. 3 *C*–*G*). In fixed (formalin-fixed, paraffin-embedded [FFPE]) autopsy samples, in 4 out of 14 patients (Fig. 3*E* and *SI Appendix*, Table S2), and in BALF samples, in 4 out of 6 patients (Fig. 3*G* and *SI Appendix*, Table S3), at least ∼20% of the viral reads were derived from negative-strand viral RNA. In contrast to acutely infected cells (Fig. 3 *C* and *D* and *SI Appendix*, Table S1), there was little or no evidence for virus reproduction in these autopsy samples (60). As summarized in *SI Appendix*, Table S2, there were negative-strand viral sequences in a large fraction of the human–viral chimeric reads (up to ∼40%) in samples from one patient. Different samples derived from the same patient revealed a similarly high fraction of negative viral strand–human RNA reads. Several other patient samples revealed lower fraction of negative viral strand RNA–human RNA chimeras, which were, however, still significantly higher than what was found in acutely infected cells (Fig. 3 *D* and *F* and *SI Appendix*, Table S1 and S2). Because the ability to identify viral–human chimeric reads using short-read RNA-seq is limited, our analysis failed to show significant numbers of chimeric reads in patient BALF samples (*SI Appendix*, Table S3). In summary, our data suggest that in some patient-derived tissues, where the total number of SARS-CoV-2 sequence-positive cells may be small, a large fraction of the viral transcripts could have been transcribed from SARS-CoV-2 sequences integrated into the host genome.

## Discussion

We present here evidence that SARS-CoV-2 sequences can be reverse-transcribed and integrated into the DNA of infected human cells in culture. For two of the integrants, we recovered “human–viral–human” chimeric reads encompassing a direct target site repeat (20 or 13 bp), and a consensus recognition site of the LINE1 endonuclease was present on both ends of the host DNA that flanked the viral sequences. These and other data are consistent with a target primed reverse transcription and retroposition integration mechanism (41, 42) and suggest that endogenous LINE1 RT can be involved in the reverse transcription and integration of SARS-CoV-2 sequences in the genomes of infected cells.

Approximately 30% of viral integrants analyzed in cultured cells lacked a recognizable nearby LINE1 endonuclease recognition site. Thus, it is also possible that integration can occur by another mechanism. Indeed, there is evidence that chimeric cDNAs can be produced in cells acutely infected with LCMV by copy choice with endogenous IAP elements during reverse transcription. This mechanism is expected to create a chimeric cDNA complementary to both LCMV and IAP. In some cases, the resulting chimeric cDNAs were integrated without the generation of a target site duplication (29). A recent study has also suggested that the interaction between coronavirus sequences and endogenous retrotransposon could be a potential viral integration mechanism (40).

It will be important, in follow-up studies, to demonstrate the presence of SARS-CoV-2 sequences integrated into the host genome in patient tissues. However, this will be technically challenging because only a small fraction of cells in any patient tissues are expected to be positive for viral sequences (61). Consistent with this notion, it has been estimated that only between 1 in 1,000 and 1 in 100,000 mouse cells infected with LCMV either in culture or in the animal carried viral DNA copies integrated into the genome (30). In addition, only a fraction of patients may carry SARS-CoV-2 sequences integrated in the DNA of some cells. However, with more than 140 million humans infected with SARS-CoV-2 worldwide (as of April, 2021), even a rare event could be of significant clinical relevance. It is also challenging to estimate the frequency of retro-integration events in cell culture assays since infected cells usually die and are lost before sample collection. For the same reason, no clonal expansion of integrated cells is expected in acute infection experiments. Moreover, the chance of integration at the same genomic locus in different patients/tissues may be low, due to a random integration process.

The presence of chimeric virus–host RNAs in cells cannot alone be taken as strong evidence for transcription of integrated viral sequences because template switching can happen during the reverse transcription step of cDNA library preparation. However, we found that only a very small fraction (0–1%) of chimeric reads from acutely infected cells contained negative-strand viral RNA sequences, whereas, in the RNA-seq libraries prepared from some patients, the fraction of total viral reads, and the fraction of human–viral chimeric reads that were derived from negative-strand SARS-CoV-2 RNAs was substantially higher. For retrotransposon-mediated integration events, the orientation of the reverse-transcribed SARS-CoV-2 RNA should be random with respect to the orientation of a host gene. Thus, for chimeric RNAs derived from integrated viral sequences, about half of the chimeric reads will link positive-strand host RNA sequences to negative-strand viral sequences. In some patient samples, negative-strand viral reads accounted for 40–50% of the total viral RNA sequences and a similar fraction of the chimeric reads contained negative-strand viral RNA sequences, suggesting that the majority if not all of the viral RNAs in these samples were derived from integrated viral sequences.

It is important to note that, because we have detected only subgenomic sequences derived mainly from the 3′ end of the viral genome integrated into the DNA of the host cell, infectious virus cannot be produced from such integrated subgenomic SARS-CoV-2 sequences. The possibility that SARS-CoV-2 sequences can be integrated into the human genome and expressed in the form of chimeric RNAs raises several questions for future studies. Do integrated SARS-CoV-2 sequences express viral antigens in patients and might these influence the clinical course of the disease? The available clinical evidence suggests that, at most, only a small fraction of the cells in patient tissues express viral proteins at a level that is detectable by immunohistochemistry. However, if a cell with an integrated and expressed SARS-CoV-2 sequences survives and presents a viral- or neo-antigen after the infection is cleared, this might engender continuous stimulation of immunity without producing infectious virus and could trigger a protective response or conditions such as autoimmunity as has been observed in some patients (62, 63). The presence of LCMV sequences integrated in the genomes of acutely infected cells in mice led the authors to speculate that expression of such sequences “potentially represents a naturally produced form of DNA vaccine” (30). It is not known how many antigen-presenting cells are needed to elicit an antigen response, but derepressed LINE1 expression, induced by viral infection or by exposure to cytokines (38–40), may stimulate SARS-CoV-2 integration into the genome of infected cells in patients. More generally, our results suggest that integration of viral DNA in somatic cells may represent a consequence of a natural infection that could play a role in the effects of other common disease-causing RNA viruses such as dengue, Zika, or influenza virus.

Our results may also be relevant for current clinical trials of antiviral therapies (64). If integration and expression of viral RNA are fairly common, reliance on extremely sensitive PCR tests to determine the effect of treatments on viral replication and viral load may not always reflect the ability of the treatment to fully suppress viral replication because the PCR assays may detect viral transcripts that derive from viral DNA sequences that have been stably integrated into the genome rather than infectious virus.

## Materials and Methods

### Cell Culture and Plasmid Transfection.

HEK293T cells were obtained from ATCC (CRL-3216) and cultured in DMEM supplemented with 10% heat-inactivated FBS (HyClone; SH30396.03) and 2 mM l-glutamine (MP Biomedicals; IC10180683) following ATCC’s method. Calu3 cells were obtained from ATCC (HTB-55) and cultured in EMEM (ATCC; 30-2003) supplemented with 10% heat-inactivated FBS (HyClone; SH30396.03) following ATCC’s method.

Plasmids for human LINE1 expression, pBS-L1PA1-CH-mneo (CMV-LINE-1), was a gift from Astrid Roy-Engel, Tulane University Health Sciences Center, New Orleans, LA (Addgene plasmid #51288 ; http://addgene.org/51288; RRID:Addgene_51288) (65); EF06R (5′UTR-LINE-1) was a gift from Eline Luning Prak, University of Pennsylvania, Philadelphia, PA (Addgene plasmid #42940 ; http://addgene.org/42940; RRID:Addgene_42940) (66). Transfection was done with Lipofectamine 3000 (Invitrogen; L3000001) following manufacturer’s protocol.

### SARS-CoV-2 Infection.

SARS-CoV-2 USA-WA1/2020 (GenBank: MN985325.1) was obtained from BEI Resources and expanded and tittered on Vero cells. Cells were infected in DMEM plus 2% FBS for 48 h using a multiplicity of infection (MOI) of 0.5 for infection of HEK293T cells and an MOI of 1 or 2 for Calu3 cells. All sample processing and harvest with infectious virus were done in the BSL3 facility at the Ragon Institute.

### Nucleic Acids Extraction and PCR Assay.

Cellular DNA extraction was done using a published method (31). For purification of genomic DNA, total cellular DNA was fractionated on a 0.4% (wt/vol) agarose/1× TAE gel for 1.5 h with a 3 V/cm voltage, with λ DNA-HindIII Digest (NEB; N3012S) as size markers. Large fragments (>23.13 kb) were cut out, frozen in −80 °C, and then crushed with a pipette tip. Three volumes (vol/wt) of high T-E buffer (10 mM Tris–10 mM EDTA, pH 8.0) were added, and then NaCl was added to give a final concentration of 200 mM. The gel solution was heated at 70 °C for 15 min with constant mixing and then extracted with phenol:chloroform:isoamyl alcohol (25:24:1, vol/vol/vol) (Life Technologies; 15593031) and chloroform:isoamyl alcohol 24:1 (Sigma; C0549-1PT). DNA was precipitated by the addition of sodium acetate and isopropyl alcohol. For samples with low DNA concentration, glycogen (Life Technologies; 10814010) was added as a carrier to aid precipitation.

RNA extraction was done with RNeasy Plus Micro Kit (Qiagen; 74034) following manufacturer’s protocol.

To detect DNA copies of SARS-CoV-2 sequences, we chose four NC gene-targeting PCR primer sets that are used in COVID-19 tests [*SI Appendix*, Fig. S1*A*, primer source from World Health Organization (67), modified to match the genome version of NC_045512.2]. See *SI Appendix*, Table S4 for PCR primer sequences used in this study. PCR was done using AccuPrime Taq DNA Polymerase, high fidelity (Life Technologies; 12346094). PCR products were run on 1% or 2% (wt/vol) agarose gel to show amplifications.

### Nanopore DNA Sequencing and Analysis.

A total of 1.6 μg of DNA extracted from HEK293T cells transfected with the pBS-L1PA1-CH-mneo (CMV-LINE-1) plasmid and infected with SARS-CoV-2 was used to make a sequencing library with the SQK-LSK109 kit (Oxford Nanopore Technologies) and sequenced on one R9 PromethION flowcell (FLO-PRO002) for 3 d and 5 min. The sequencing data were base-called using Guppy 4.0.11 (Oxford Nanopore Technologies) using the high-accuracy model.

Nanopore reads were mapped using minimap2 (68) (version 2.15) with parameters “-p 0.3 -ax map-ont” and a fasta file containing the human genome sequence from ENSEMBL release 93 (ftp://ftp.ensembl.org/pub/release-93/fasta/homo_sapiens/dna/Homo_sapiens.GRCh38.dna.primary_assembly.fa.gz) concatenated to the SARS-CoV-2 sequence, GenBank ID: MN988713.1, “Severe acute respiratory syndrome coronavirus 2 isolate SARS-CoV-2/human/USA/IL-CDC-IL1/2020, complete genome.” From the SAM file, we selected all the sequences that mapped to the viral genome and divided them into groups based on the human chromosomes they mapped to. We blasted the selected sequences, using blastn, against a BLAST database made with the human and virus sequences described above. We parsed the blast output into a text file containing one row per high-scoring segment pair (HSP) with a custom perl script. We further filtered that file, for each sequence, by selecting all the viral HSPs and the top three human HSPs. We inspected those files visually to identify sequences containing human–viral–human or human–viral junctions. For a few sequences, longer than 30 kb, we inspected the top 15 human HSPs. Additionally, we visually inspected all the identified reads containing human and viral sequences by the University of California, Santa Cruz (UCSC) BLAT (69) tool. Due to errors in Nanopore sequencing and/or base-calling, artifactual “hybrid sequences” exist in a subset of these reads, sometimes with Watson and Crick strands from the same DNA fragment present in the same read. Therefore, we only focused on chimeric sequences showing clear human–viral junctions and analyzed known LINE1-mediated retroposition features such as target-site duplications and LINE1 endonuclease recognition sequences for evidence of integration.

### Tn5 Tagmentation-Mediated Integration Site Enrichment.

We used a tagmentation-based method to enrich for viral integration sites (47, 48). Briefly, we used Tn5 transposase (Diagenode; C01070010) to randomly tagment the cellular DNA with adapters (adapter A, the Illumina Nextera system). Tagmentation was done using 100 ng of DNA for 10 min at 55 °C, followed by stripping off the Tn5 transposase from the DNA with SDS. We used a reverse primer targeting the near-5′ end of SARS-CoV-2 NC gene (CCA​AGA​CGC​AGT​ATT​ATT​GGG​TAA​A) or a forward primer targeting the near-3′ end of SARS-CoV-2 genome (CTT​GTG​CAG​AAT​GAA​TTC​TCG​TAA​CT) to linearly amplify (PCR0, 45 cycles) the tagmented DNA fragments containing viral sequences. We took the product of PCR0 and amplified the DNA fragments containing adapter and viral sequences (potential integration sites) using 15–20 cycles of PCR1, with a barcoded (i5) Nextera primer (AAT​GAT​ACG​GCG​ACC​ACC​GAG​ATC​TAC​ACN​NNN​NNN​NTC​GTC​GGC​AGC​GTC, NNNNNNNN indicates the barcode) against the adapter sequence and a viral primer. The viral primer was designed to either target the near-5′ end of SARS-CoV-2 NC gene (GTC​TCG​TGG​GCT​CGG​AGA​TGT​GTA​TAA​GAG​ACA​GGCC​GAC​GTT​GTT​TTG​ATC​G, viral sequence underlined) or target the near-3′ end of SRAS-CoV-2 genome (GTC​TCG​TGG​GCT​CGG​AGA​TGT​GTA​TAA​GAG​ACA​GCGC​GGA​GTA​CGA​TCG​AGT​G, viral sequence underlined). The viral primer also contained an adapter sequence for further PCR amplification. We amplified the PCR1 product by 15–20 cycles of PCR2, using a short primer (AAT​GAT​ACG​GCG​ACC​ACC​GA) against the i5 Nextera primer sequence and a barcoded (i7) Nextera primer (CAA​GCA​GAA​GAC​GGC​ATA​CGA​GAT​NNN​NNN​NNG​TCT​CGT​GGG​CTC​GG, NNNNNNNN indicates the barcode) against the adapter sequence introduced by the viral primer in PCR1. The final product of the PCR2 amplification was fractionated on 1.5% agarose gel (Sage Science; HTC1510) with PippinHT (Sage Science; HTP0001) and 500- to 1,000-bp pieces were selected for Illumina paired-end sequencing. All three PCR steps (PCR0–PCR2) were done with KAPA HiFi HotStart ReadyMix (KAPA;KK2602).

### Illumina DNA Sequencing and Analysis.

We constructed libraries for HEK293T cell whole-genome sequencing using the Tn5-based Illumina DNA Prep kit (Illumina; 20018704). The whole-genome sequencing libraries or the libraries from Tn5-mediated integration site enrichment after sizing (described above) were subjected to Illumina sequencing. qPCR was used to measure the concentrations of each library using KAPA qPCR library quant kit according to the manufacturer’s protocol. Libraries were then pooled at equimolar concentrations, for each lane, based on qPCR concentrations. The pooled libraries were denatured using the Illumina protocol. The denatured libraries were loaded onto an SP flowcell on an Illumina NovaSeq 6000 and run for 2 × 150 cycles according to the manufacturer’s instructions. Fastq files were generated and demultiplexed with the bcl2fastq Conversion Software (Illumina).

To identify human–SARS-CoV-2 chimeric DNA reads, raw sequencing reads were aligned with STAR (70) (version 2.7.1a) to a human plus SARS-CoV-2 genome made with a fasta file containing the human genome sequence version hg38 with no alternative chromosomes concatenated to the SARS-CoV-2 sequence from National Center for Biotechnology Information (NCBI) reference sequence NC_045512.2. The following STAR parameters were used to call chimeric reads: –alignIntronMax 1 \–chimOutType Junctions SeparateSAMold WithinBAM HardClip \–chimScoreJunctionNonGTAG 0 \–alignSJstitchMismatchNmax -1–1 -1–1 \–chimSegmentMin 25 \–chimJunctionOverhangMin 25 \–outSAMtype BAM SortedByCoordinate. We extracted viral reads from the generated BAM file by samtools (71) (version 1.11) using command: samtools view -b Aligned.sortedByCoord.out.bam NC_045512v2 > NC_Aligned.sortedByCoord.out.bam. We extracted human–viral chimeric reads by using the read names from the STAR generated Chimeric.out.junction file to get the read alignments from the STAR generated Chimeric.out.sam file by Picard (http://broadinstitute.github.io/picard), using command: java -jar picard.jar FilterSamReads I = Chimeric.out.sam O = hv-Chimeric.out.sam READ_LIST_FILE = hv-Chimeric.out.junction.ids FILTER = includeReadList. We further confirmed each of the chimeric reads and filtered out any unconvincing reads (too short or aligned to multiple sites of the human genome) by visual inspection with the UCSC BLAT (69) tool. We also loaded the STAR generated Aligned.sortedByCoord.out.bam file or the NC_Aligned.sortedByCoord.out.bam file containing extracted viral reads to the UCSC browser SARS-CoV-2 genome (NC_045512.2) to search for additional chimeric reads that were missed by the STAR chimeric calling method. To generate genome coverage file, we used the bamCoverage from the deepTools suite (72) (version 3.5.0) to convert the STAR generated Aligned.sortedByCoord.out.bam file to a bigwig file binned at 10 bp, using command: bamCoverage -b Aligned.sortedByCoord.out.bam -o Aligned.sortedByCoord.out.bw–binSize 10.

### RNA-Seq and Analysis.

To identify human–SARS-CoV-2 chimeric reads, published RNA-seq data were downloaded from Gene Expression Omnibus (GEO) with the accession numbers GSE147507 (50), GSE153277 (51), GSE156754 (52), GSE157852 (53), GSE153684 (54), and GSE154998 (55) (summarized in *SI Appendix*, Fig. S5*C*). Raw sequencing reads were aligned with STAR (70) (version 2.7.1a) to human plus SARS-CoV-2 genome and transcriptome made with a fasta file containing the human genome sequence version hg38 with no alternative chromosomes concatenated to the SARS-CoV-2 sequence from NCBI reference sequence NC_045512.2, and a gtf file containing the human gene annotations from ENSEMBL version GRCh38.97 concatenated to the SARS-CoV-2 gene annotations from NCBI (http://hgdownload.soe.ucsc.edu/goldenPath/wuhCor1/bigZips/genes/). The following STAR parameters (56) were used to call chimeric reads unless otherwise specified (*SI Appendix*, Fig. S5*C*):–chimOutType Junctions SeparateSAMold WithinBAM HardClip \–chimScoreJunctionNonGTAG 0 \–alignSJstitchMismatchNmax -1–1 -1–1 \–chimSegmentMin 50 \–chimJunctionOverhangMin 50.

For RNA-seq strandedness analysis, we generated RNA-seq data using RNA from SARS-CoV-2–infected Calu3 cells. Stranded libraries were constructed with the Kapa mRNA HyperPrep kit (Roche; 08098115702). Libraries were qPCR'ed using a KAPA qPCR library quant kit as per manufacturer’s protocol. Libraries were then pooled at equimolar concentrations, for each lane, based on qPCR concentrations. The pooled libraries were denatured using the Illumina protocol. The denatured libraries were loaded onto an HiSeq 2500 (Illumina) and sequenced for 120 cycles from one end of the fragments. Basecalls were performed using Illumina offline basecaller (OLB) and then demultiplexed. We downloaded published RNA-seq data (stranded libraries) from GEO with the accession numbers GSE147507 (50) (Calu3, *SI Appendix*, Table S1), GSE148697 (58) (lung organoids, *SI Appendix*, Table S1), and GSE150316 (60) (patient FFPE tissues, *SI Appendix*, Table S2**).** Raw RNA-seq reads were aligned as described above, using parameters–chimSegmentMin 30 \–chimJunctionOverhangMin 30 to call chimeric reads. We extracted total viral reads and human–viral chimeric reads as described above. We convert the viral read BAM files into Bed files using the bamToBed utility in BEDTools (73). We then counted the total and stranded read numbers in the converted BED files.

Published single-cell RNA-seq data were downloaded from GEO with the accession number GSE145926 (61) (patient BALF samples, *SI Appendix*, Table S3). For bulk analysis, duplicate reads with the same read1 (UMI) and read2 sequences in raw fastq files were removed by dedup_hash (https://github.com/mvdbeek/dedup_hash). Then the pool of read2 were aligned as described above, using parameters –chimSegmentMin 30 \–chimJunctionOverhangMin 30 to call chimeric reads. Read strandedness was analyzed as described above. For single-cell analysis, we generated a custom genome by Cell Ranger (10× Genomics Cell Ranger 3.0.2) (74) mkref, using a fasta file containing the human genome sequence from ENSEMBL release 93 (ftp://ftp.ensembl.org/pub/release-93/fasta/homo_sapiens/dna/Homo_sapiens.GRCh38.dna.primary_assembly.fa.gz) concatenated to the SARS-CoV-2 sequence, GenBank ID: MN988713.1, and a gtf file containing human and viral annotations. Read mapping, assigning reads to cell barcodes and removing PCR duplicates were done with Cell Ranger (10× Genomics Cell Ranger 4.0.0) (74) count, using the custom genome described above. We processed the counts using Seurat (version 3.2.2) (75). We removed cells that had less than 200 genes detected or more than 20% of transcript counts deriving from the mitochondria. For each cell, we counted the number of reads mapping to either the positive or negative viral strand.

## Supplementary Material

Supplementary File

Supplementary File

Supplementary File

Supplementary File

Supplementary File

## Data Availability

All data supporting the findings of this study are available within the article and supporting information. All sequencing data generated in this study have been deposited to the Sequence Read Archive, https://www.ncbi.nlm.nih.gov/sra (accession no. PRJNA721333). All published data analyzed in this study are cited in this article with accession methods provided in *Materials and Methods*.

## Change History

July 14, 2021: The SI appendix has been updated.
