# Supplementary material for: Reverse-transcribed SARS-CoV-2 RNA can integrate into the genome of cultured human cells and can be expressed in patient-derived tissues
Source: Proc Natl Acad Sci U S A. 2021 May 6;118(21):e2105968118. doi: 10.1073/pnas.2105968118 (PMC8166107; doi:10.1073/pnas.2105968118)
Supplement: Supplementary File [file pnas.2105968118.sapp.pdf]

PNAS
www.pnas.org

Supplementary Information for

Reverse-transcribed SARS-CoV-2 RNA can integrate into the genome of cultured human cells and can be expressed in patient-derived tissues

Liguo Zhang[1], Alexsia Richards[1], M. Inmaculada Barrasa[1], Stephen H. Hughes[2], Richard A. Young[1, 3] and Rudolf Jaenisch[1, 3, #]

[1]Whitehead Institute for Biomedical Research, Cambridge, MA, USA.

[2] HIV Dynamics and Replication Program, Center for Cancer Research, National Cancer Institute, Frederick, MD, USA.

[3]Department of Biology, Massachusetts Institute of Technology, Cambridge, MA, USA.

[#]Correspondence: jaenisch@wi.mit.edu

This PDF file includes:

Figures S1 to S7
Tables S1 to S5
Legends for Datasets S1 to S4

Other supplementary materials for this manuscript include the following:

Datasets S1 to S4

Supplementary Figures

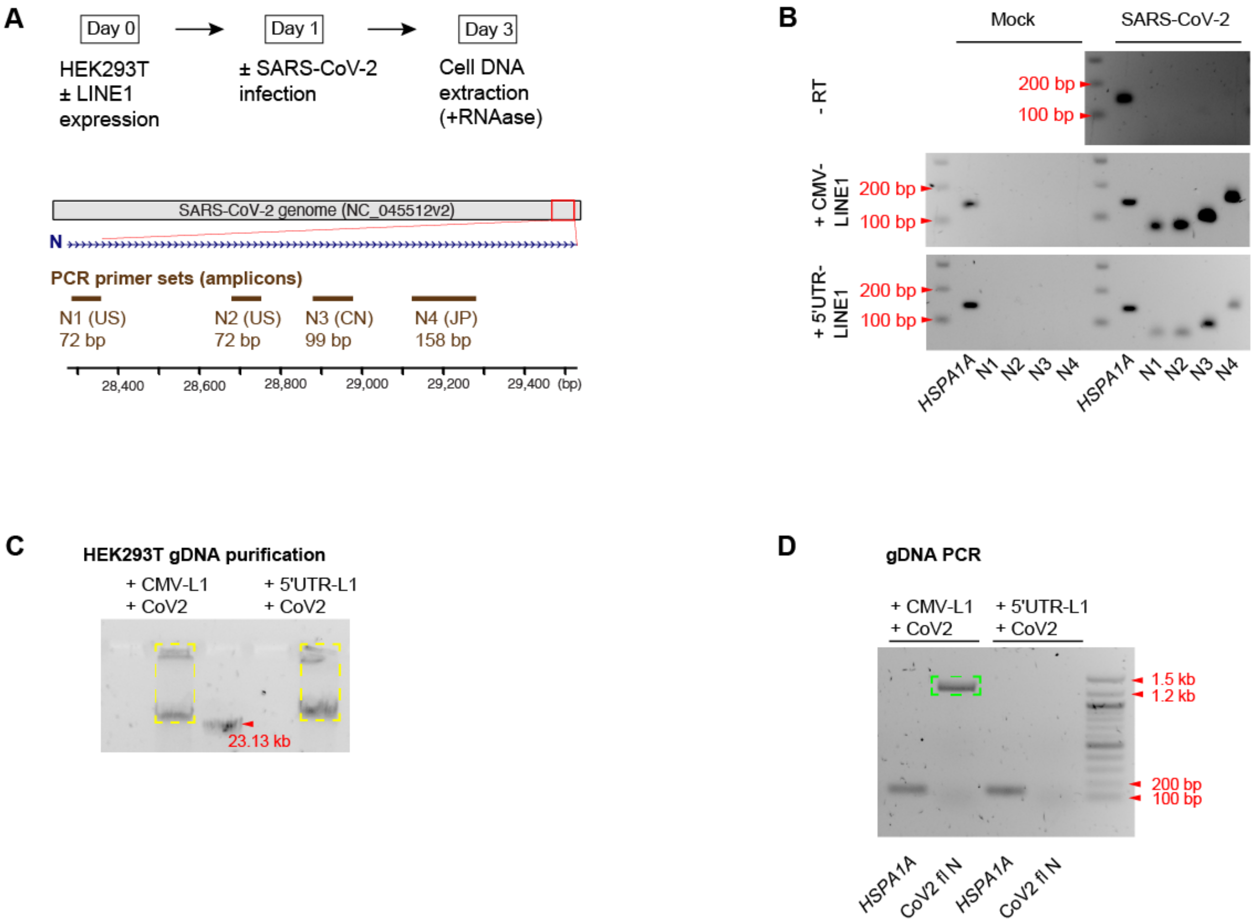

Fig. S1. Detection of DNA copies of SARS-CoV-2 RNA in infected LINE1-overexpressing cells. A) Experimental workflow (top) and PCR primer sets (bottom) used to detect reverse-transcription and integration of SARS-CoV-2 RNA. **B)** PCR detection of SARS-CoV-2 NC sequences in DNA purified from mock (left) or SARS-CoV2 (right) infected HEK293T cells without or with transfection of human LINE1 (CMV-LINE1 or 5'UTR-LINE1) plasmids. *HSPA1A*: human *HSPA1A* gene as control; N1 – N4: SARS-CoV-2 NC amplicons as shown in **A)**. N1 – N4 PCR products were loaded on gel three times the amount of *HSPA1A* PCR product. Note that we didn't detect DNA copies of SARS-CoV-2 sequences in cells without LINE1 overexpression by this low-sensitive PCR assay. **C)** Gel purification of large fragments of genomic DNA (yellow boxes) from SARS-CoV-2 infected HEK293T cells that were transfected

with CMV-LINE1 or 5'UTR-LINE1. **D)** Cloning of a DNA copy of a complete SARS-CoV-2 NC gene sequence (CoV2 fl N, green box) from gel-purified HEK293T genomic DNA**.**

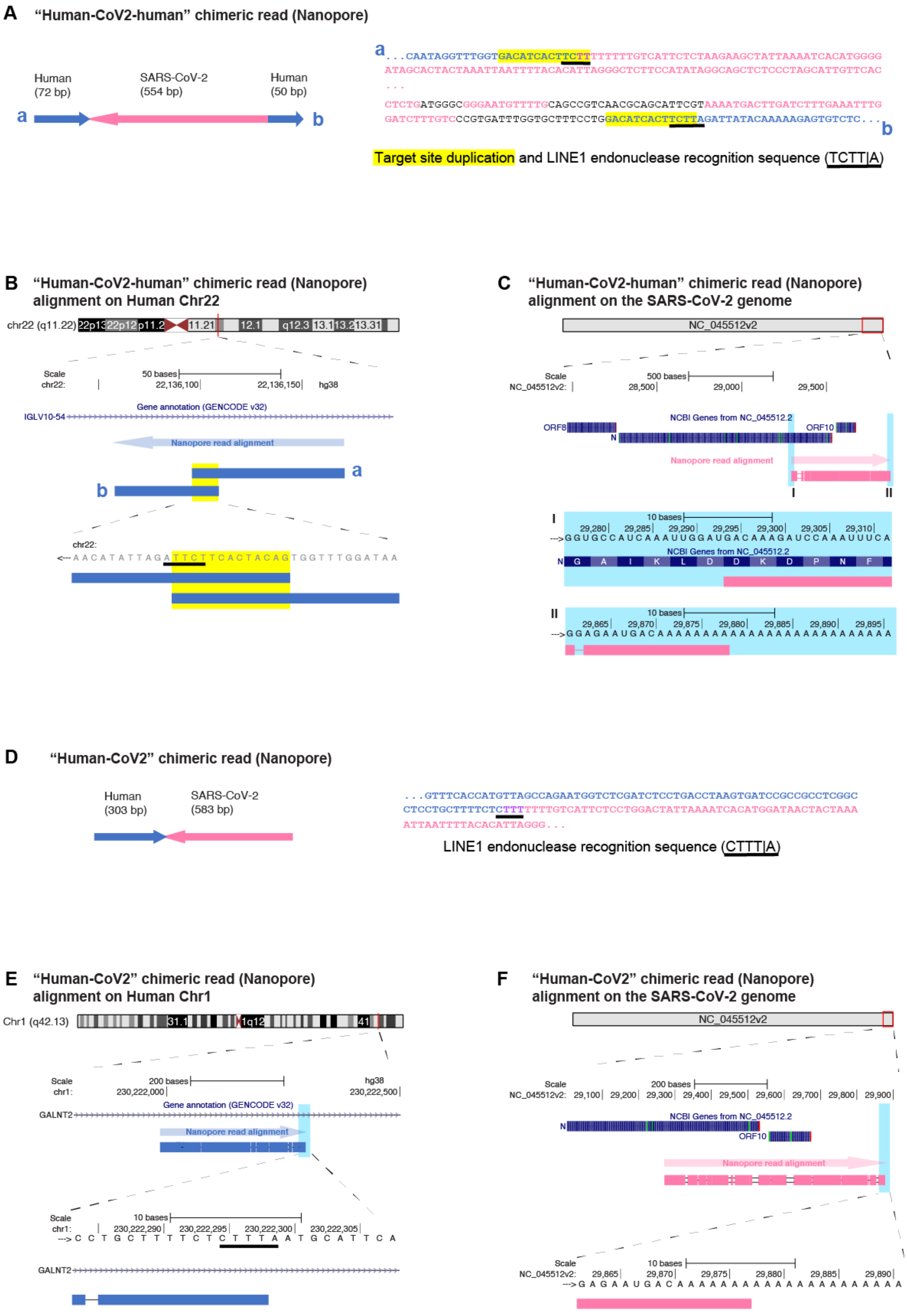
A
"Human-CoV2-human" chimeric read (Nanopore)
Human (72 bp)
SARS-CoV-2 (554 bp)
Human (50 bp)
a
b
a ...CAATAGGTTTGGTGACATCACTTCTTTTTTTTGTCATTCTCTAAGAAGCTATTAAAATCACATGGGG
ATAGCACTACTAAATTAATTTTACACATTAGGGCTCTTCCATATAGGCAGCTCTCCCTAGCATTGTTCAC
...
CTCTGATGGGCGGGAATGTTTTGCAGCCGTCAACGCAGCATTCGTAAAATGACTTGATCTTTGAAATTTG
GATCTTTGTCCCGTGATTTGGTGCTTTCCTGGACATCACTTCTTAGATTATACAAAAGAGTGTCTC... b
Target site duplication and LINE1 endonuclease recognition sequence (TCTT|A)
B
"Human-CoV2-human" chimeric read (Nanopore) alignment on Human Chr22
chr22 (q11.22)
Scale
chr22:
50 bases
22,136,100
22,136,150
hg38
Gene annotation (GENCODE v32)
IGLV10-54
Nanopore read alignment
a
b
chr22:
AACATATTAGATTCTTCACTACAGTGGTTTGGATAA
C
"Human-CoV2-human" chimeric read (Nanopore) alignment on the SARS-CoV-2 genome
NC_045512v2
Scale
NC_045512v2:
500 bases
28,500
29,000
29,500
NCBI Genes from NC_045512.2
ORF8
N
ORF10
Nanopore read alignment
I
II
10 bases
29,280
29,285
29,290
29,295
29,300
29,305
29,310
GGUGCCAUCAAAUUGGAUGACAAAGAUCCAAAUUUCA
NCBI Genes from NC_045512.2
N G A I K L D D K D P N F
10 bases
29,865
29,870
29,875
29,880
29,885
29,890
29,895
GGAGAAUGACAAAAAAAAAAAAAAAAAAAAAAAAAAA
D
"Human-CoV2" chimeric read (Nanopore)
Human (303 bp)
SARS-CoV-2 (583 bp)
...GTTTCACCATGTTAGCCAGAATGGTCTCGATCTCCTGACCTAAGTGATCCGCCGCCTCGGC
CTCCTGCTTTTCTCTTTTTTTGTCATTCTCCTGGACTATTAAAATCACATGGATAACTACTAAA
ATTAATTTTACACATTAGGG...
LINE1 endonuclease recognition sequence (CTTT|A)
E
"Human-CoV2" chimeric read (Nanopore) alignment on Human Chr1
Chr1 (q42.13)
Scale
chr1:
200 bases
230,222,000
230,222,500
hg38
Gene annotation (GENCODE v32)
GALNT2
Nanopore read alignment
Scale
chr1:
10 bases
230,222,290
230,222,295
230,222,300
230,222,305
CCTGCTTTTCTCTTTAATGCATTCA
GALNT2
F
"Human-CoV2" chimeric read (Nanopore) alignment on the SARS-CoV-2 genome
NC_045512v2
Scale
NC_045512v2:
200 bases
29,100
29,200
29,300
29,400
29,500
29,600
29,700
29,800
29,900
NCBI Genes from NC_045512.2
N
ORF10
Nanopore read alignment
Scale
NC_045512v2:
10 bases
29,865
29,870
29,875
29,880
29,885
29,890
GAGAAUGACAAAAAAAAAAAAAAAAAAAAAAA

Fig. S2. Nanopore sequencing reads provide evidence for integration of SARS-CoV-2 sequences. A) A Nanopore sequencing read showing integration of a SARS-CoV-2 NC sub-genomic RNA sequence (magenta) and human genomic sequences (blue) flanking both sides of the integrated viral sequence. Features indicative of LINE1 mediated "target-primed reverse transcription" include: the target site duplication (yellow highlight) and the LINE1 endonuclease recognition sequence (underlined). Sequences that could be aligned to both genomes are shown in purple and sequences that cannot be aligned are shown in black. Arrows indicate sequence orientations with regard to the human and SARS-CoV-2 genomes as shown in **B, C**), with the two sides labeled as "a" and "b" in blue. **B)** Alignment of the Nanopore read in **A**) with the human genome (chromosome 22) showing the integration site. The human sequences at the junction region show the target site which was duplicated when the SARS-CoV-2 cDNA was integrated (yellow highlight) and the LINE1 endonuclease recognition sequence (underlined). **C)** Alignment of the Nanopore read in **A**) with the SARS-CoV-2 genome showing the integrated viral DNA represents a DNA copy of a portion of the NC sub-genomic RNA. Light blue highlighted regions are enlarged to show the two ends of the read. **D)** A Nanopore sequencing read showing the integrated portion of a SARS-CoV-2 RNA sequence (magenta) and human genomic sequences (blue) from one side of the junction with a LINE1 endonuclease recognition sequence (underlined). Sequences that could be mapped to both genomes are shown in purple. Arrows indicate sequence orientations with regard to the human and SARS-CoV-2 genomes. **E)** Alignment of the Nanopore read in **D**) with the human genome (chromosome 1) showing the integration site. The light blue highlighted region is enlarged to show a LINE1 endonuclease recognition sequence (underlined). **F)** Alignment of the Nanopore read **D**) with the SARS-CoV-2 genome showing the integrated viral sequence. The light blue highlighted region is enlarged to show the 3' end of the viral sequence at the junction with human sequence.

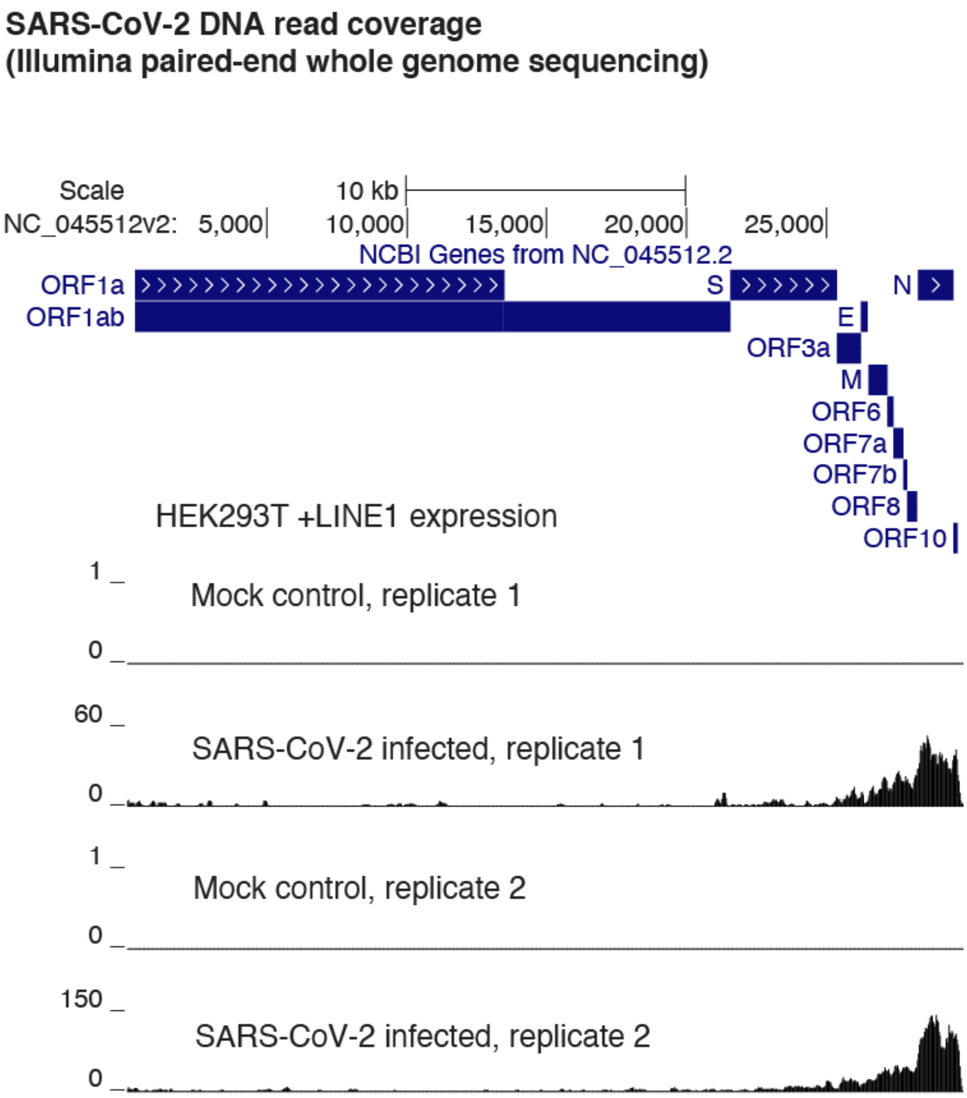

Fig. S3. Reverse transcribed viral sequences are predominant from the 3' end of SARS-CoV-2 genome. Viral reads were obtained using Illumina whole-genome paired-end sequencing of DNA from HEK293T cells that overexpressed LINE1. Genomic tracks showing the number of viral reads binned at 10 bp.

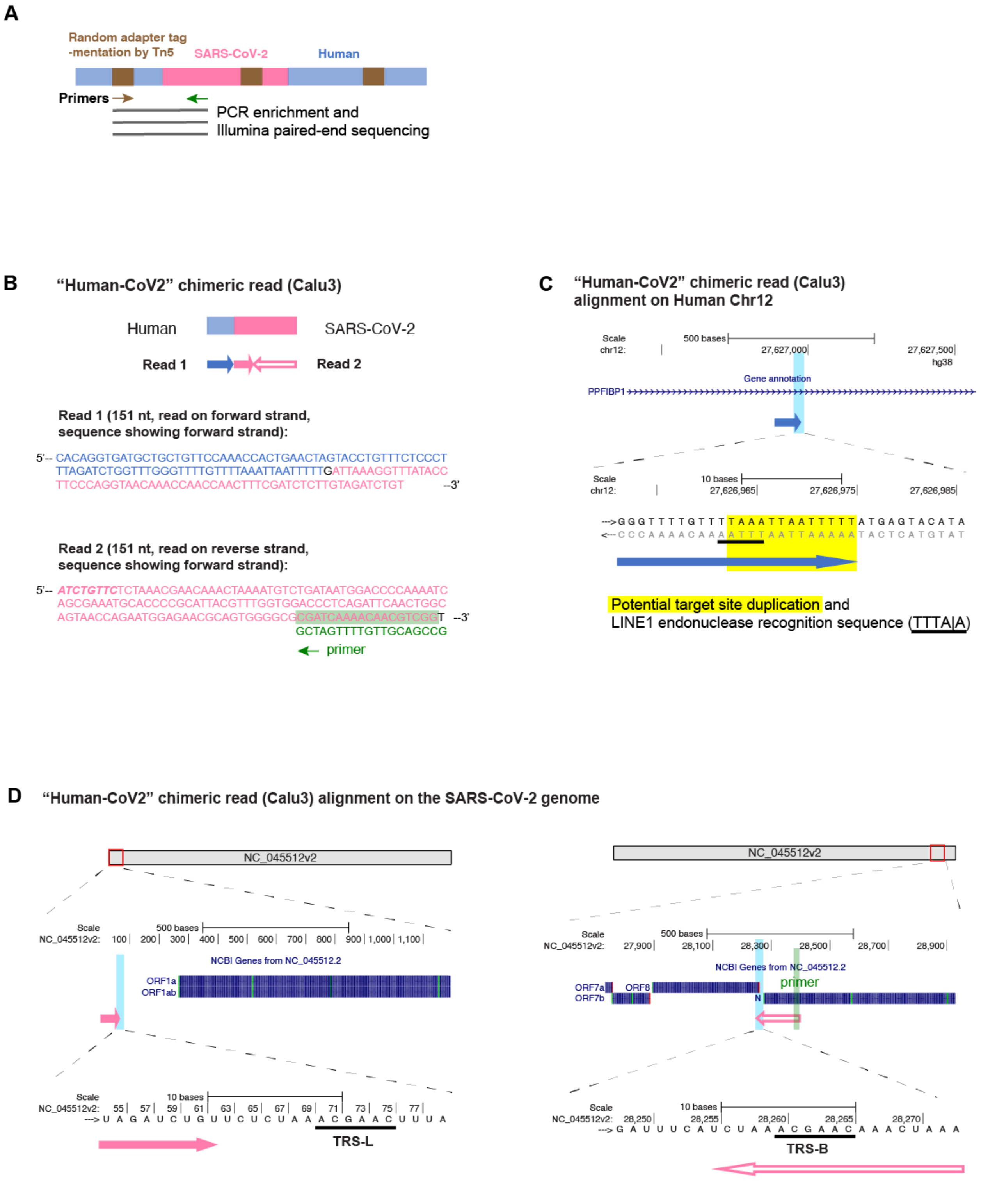

Fig. S4. Evidence for integration of SARS-CoV-2 cDNA in Calu3 cells. A) Experimental design for the Tn5 tagmentation mediated enrichment sequencing method used to map

integration sites in the host cell genome. The viral primer (reverse) was designed to target near-5' end of the viral NC gene (green arrow). **B)** A human-viral chimeric read pair supporting viral integration. The reads are aligned with the human (blue) and SARS-CoV-2 (magenta) genomic sequences. Arrows indicate read orientations relative to the human and SARS-CoV-2 genomes as shown in **C, D**). Closed arrows show read 1 in the pair that was mapped to both human (blue) and SARS-CoV-2 (magenta) sequences. The open arrow (magenta) shows read 2 in the pair that was mapped to the SARS-CoV-2 genome. The sequence corresponding to the viral primer in read 2 is shown with green highlight (corresponding to the green arrow illustrated in **A**). **C)** Alignment of the read pair in **B**) with the human genome (chromosome 12, blue arrow). The highlighted (light blue) region of the human sequence is enlarged to show the LINE1 recognition sequence (underlined) and the potential target site duplication (highlighted in yellow) that would be generated by LINE1 mediated retroposition. **D)** Alignment of the read pair in **B**) with the SARS-CoV-2 genome (magenta arrows). The closed arrow (left) corresponds to read 1 in **B**), aligned to the viral leader sequence. The open arrow (right) corresponds to read 2 in **B**), aligned to the NC gene body (the beginning 8 bases in this read is aligned to the viral leader sequence, shown in italics in **B**). The highlighted (light blue) regions of the SARS-CoV-2 sequences are enlarged to show the TRS-L (left) and TRS-B (right) sequences (underlined, these are the sequences where the viral polymerase jumps to generate the sub-genomic RNA). The viral primer sequence is shown with green highlight.

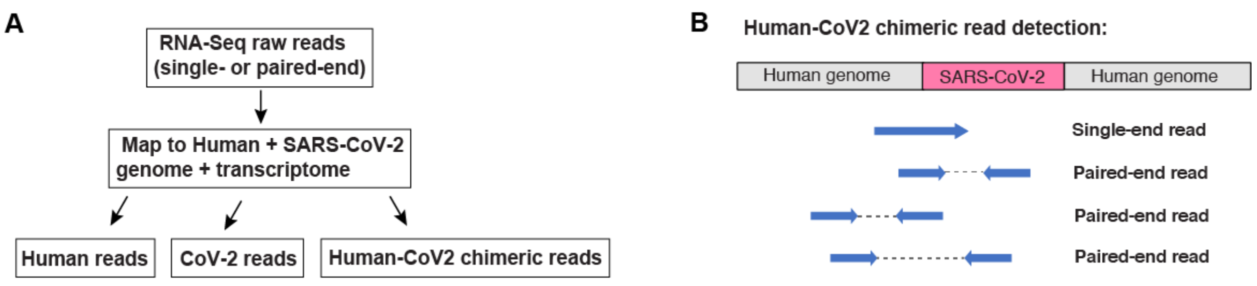

C

Data ID (GEO)	Sample	SARS-CoV-2 infection	RNA-Seq read length (nt)	Min overhang (threshold)
GSE147507	Calu3, A549-ACE2, NHBE	MOI 2, 24 hrs	150	50
GSE153277	iPSC-derived type II alveolar epithelial-like cells	MOI 5, 4 days	76+76	50
GSE156754	iPSC-derived cardiomyocytes	MOI 0.1, 48 hrs	76+76	50
GSE157852	iPSC-derived choroid plexus organoids	MOI 0.1-0.05, 72 hrs	72	20
GSE153684	Cultured organoid from pediatric stomach tissue (age 11 year)	MOI 1	51+51	20
GSE154998	Blood buffy coat cells	COVID19+ patient (ICU)	72	20

Fig. S5. Analysis of published data for human-viral chimeric transcripts. A) The pipeline used to identify human-CoV2 chimeric RNA-Seq reads. **B)** Schema showing human- SARS-CoV2 chimeric RNA-seq reads mapped to potential SARS-CoV-2 integration sites. **C)** Published data used to identify human-viral chimeric reads: Data ID (GEO accession number), sample type, infection method/type (MOI: Multiplicity of Infection), RNA-Seq format (single or paired-end with read length), and threshold to call chimeric reads (Min overhang: minimum number of bases mapped to either human or SARS-CoV-2 genome/transcriptome to call a chimeric reads).

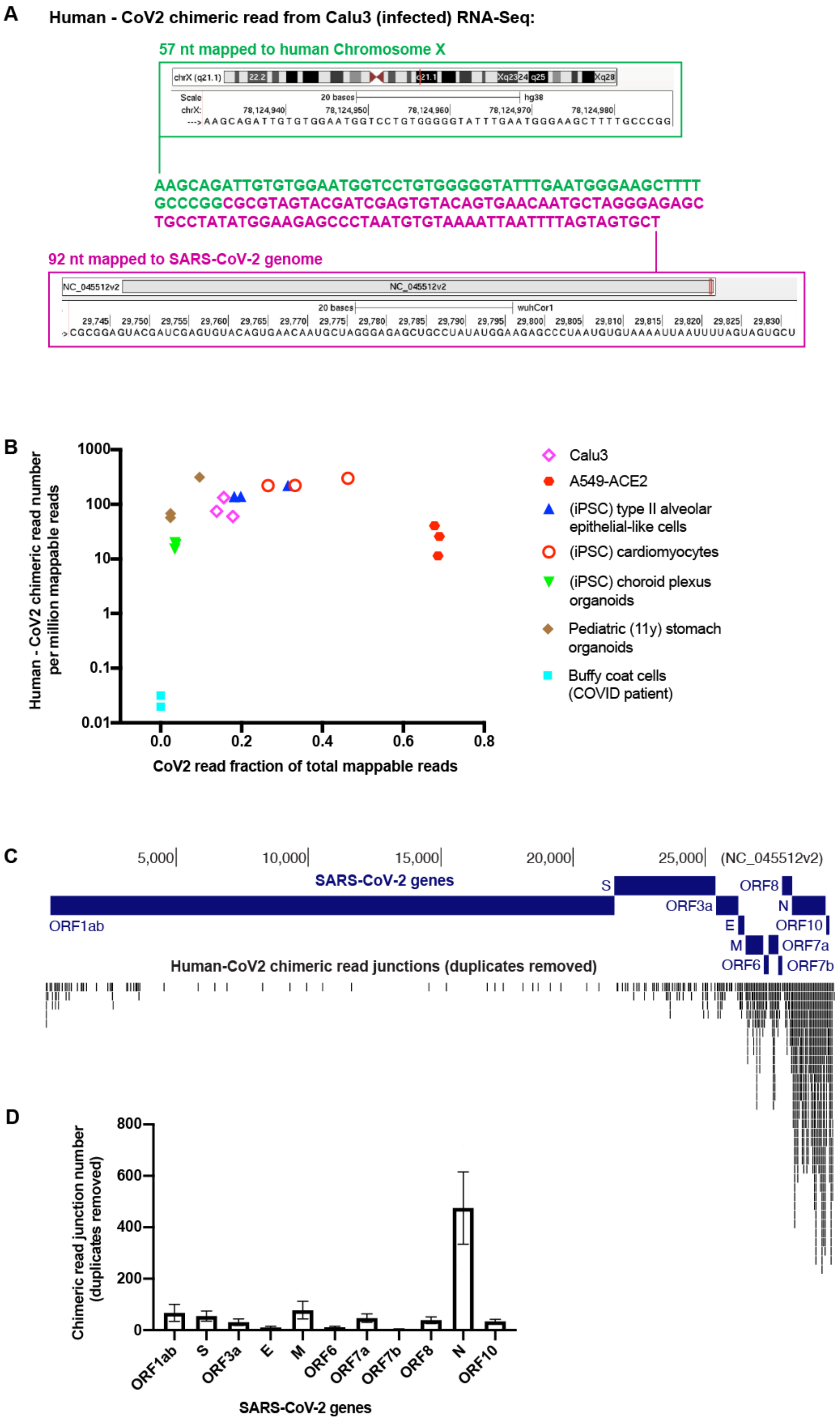

A
Human - CoV2 chimeric read from Calu3 (infected) RNA-Seq:
57 nt mapped to human Chromosome X
AAGCAGATTGTGTGGAATGGTCCTGTGGGGGTATTTGAATGGGAAGCTTTT
GCCCGGCGCGTAGTACGATCGAGTGTACAGTGAACAATGCTAGGGAGAGC
TGCCTATATGGAAGAGCCCTAATGTGTAAAATTAATTTTAGTAGTGCT
92 nt mapped to SARS-CoV-2 genome
B
Human - CoV2 chimeric read number per million mappable reads
CoV2 read fraction of total mappable reads
Calu3
A549-ACE2
(iPSC) type II alveolar epithelial-like cells
(iPSC) cardiomyocytes
(iPSC) choroid plexus organoids
Pediatric (11y) stomach organoids
Buffy coat cells (COVID patient)
C
(NC_045512v2)
SARS-CoV-2 genes
ORF1ab
S
ORF3a
E
M
ORF6
ORF7a
ORF7b
ORF8
N
ORF10
Human-CoV2 chimeric read junctions (duplicates removed)
D
Chimeric read junction number (duplicates removed)
SARS-CoV-2 genes

Fig. S6. Human-viral chimeric reads from published RNA-seq data. A) A chimeric RNA read (149 nt) from (SARS-CoV-2) infected Calu3 RNA-Seq with 57 nt mapped to human Chromosome X (green) and 92 nt (magenta) mapped to the SARS-CoV-2 genome. **B)** Scatter plot showing the number of human-CoV2 chimeric reads (per million total mappable reads, y-axis) versus the fraction of SARS-CoV-2 reads in total mappable reads (x-axis) in published RNA-Seq datasets from different SARS-CoV-2 infected samples. **C-D)** Human-CoV2 chimeric read junctions (duplicates removed) mapped to the SARS-CoV-2 genome (**C**) and distribution among SARS-CoV-2 genes (**D**, three biological replicates; mean ± s.e.m.). RNA-Seq data is from SARS-CoV-2 infected Calu3 cells (GSE147507). A chimeric read junction is defined by the "break point" of the sequences mapped to human or SARS-CoV-2 genome/transcriptome in a given RNA-Seq read.

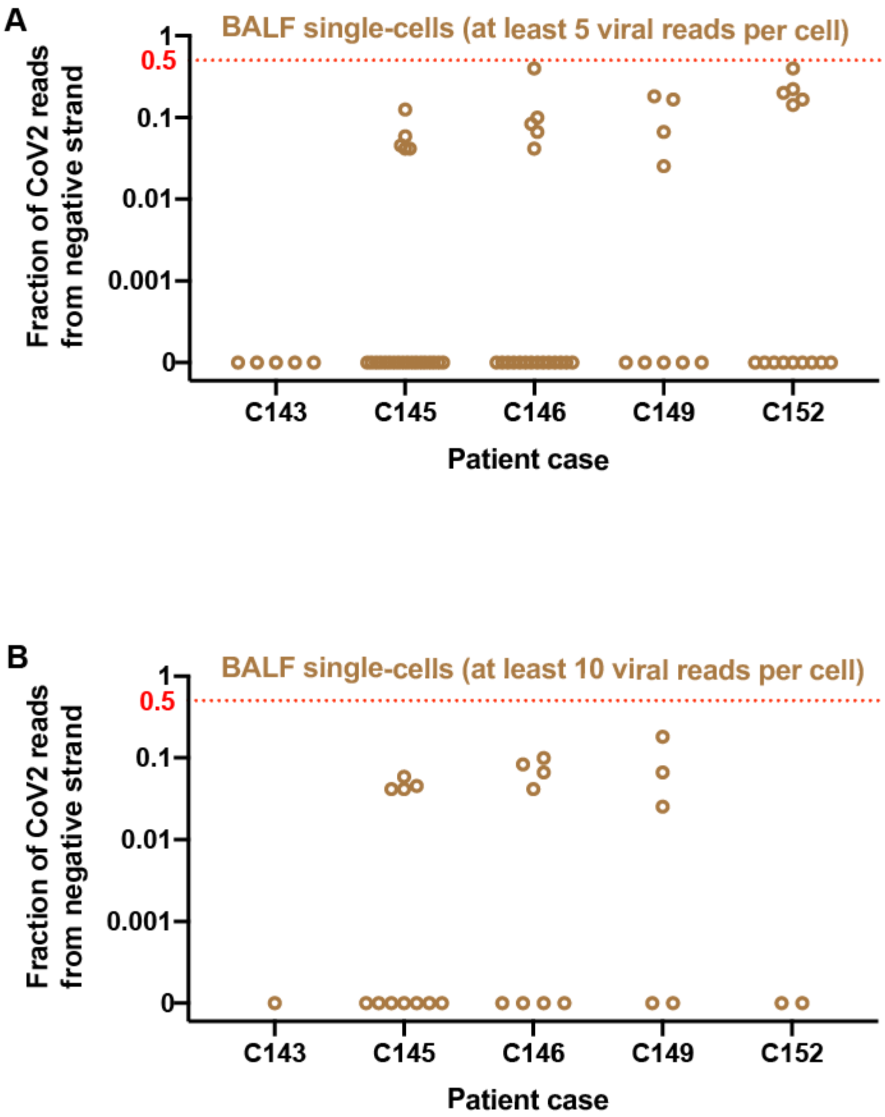

Fig. S7. Negative strand viral RNA-seq reads from patient single-cells suggest that integrated SARS-CoV-2 sequences are expressed. A) Fraction of viral reads that are derived from negative strand viral RNA in single BALF cells from patients with at least 5 viral reads per cell (published RNA-seq data, GSE145926). **B**) Fraction of viral reads that are derived from negative strand viral RNA in patient single BALF cells with at least 10 viral reads per cell (published RNA-seq data, GSE145926). Red dashed lines indicate the level at which 50% of all viral reads were from negative strand viral RNAs, a level expected if all the viral sequences were derived from integrated sequences.

Supplementary Tables

Table S1. Summary of negative strand viral and human-viral chimeric RNA-seq reads from acutely infected lung cells or organoids

Sample	CoV2 reads	Negative strand CoV2 read fraction	Human-CoV2 chimeric reads	Negative strand CoV2 (in chimeric RNAs) read fraction
Calu3, rep1	52,962,587	0.08%	8,170	0.81%
Calu3, rep2	61,256,542	0.10%	6,218	0.76%
Calu3, rep1 (Blanco-Melo et al.)	32,10,542	0.01%	4,430	0
Calu3, rep2 (Blanco-Melo et al.)	2,378,641	0.01%	1,859	0
Calu3, rep3 (Blanco-Melo et al.)	4,320,681	0.01%	9,702	0.01%
Lung organoid, rep1 (Han et al.)	615	0	1	0
Lung organoid, rep2 (Han et al.)	12,752	0.04%	15	0
Lung organoid, rep3 (Han et al.)	1,320	0.08%	2	0

Table S2. Summary of negative strand viral and human-viral chimeric RNA-seq reads from tissues of deceased COVID-19 patients (published RNA-seq data, Desai et al., GSE150316)

Sample	GEO accession number	CoV2 reads	Negative strand CoV2 read fraction	Human-CoV2 chimeric reads	Negative strand CoV2 (in chimeric RNAs) read fraction
Case1-lung1 LUL	GSM4546576	51,4418	6.7%	108	1.9%
Case1-lung2 RML	GSM4546577	54,598	8.4%	9	0.0%
Case1-lung3 RUL	GSM4546578	20,746	13.0%	6	0.0%
Case1-lung4 LLL	GSM4546579	37,232	2.4%	4	0.0%
Case2-lung1 RLL	GSM4546581	483	12.4%	0	
Case2-lung2 LUL	GSM4546582	42	0.0%	0	
Case2-jejunum1	GSM4546583	10	0.0%	0	
Case2-lung3 RUL	GSM4546584	10	0.0%	0	
Case3-lung1 LUL	GSM4546586	16	25.0%	0	
Case3-lung2 RLL	GSM4546588	4	0.0%	0	
Case5-lung1 LLL	GSM4546596	220	0.0%	0	
Case5-lung2 RML	GSM4546597	38	47.4%	0	
Case5-lung3 LUL	GSM4546598	648	0.9%	0	
Case5-lung4 RML	GSM4546599	514	0.4%	0	
Case5-lung5 RUL	GSM4546601	722	4.4%	0	
Case5-liver1	GSM4546604	6	0.0%	0	
Case6-lung1 LUL	GSM4698531	14	0.0%	0	
Case7-lung5 LUL	GSM4698540	1,284	0.6%	0	
Case8- bowel1	GSM4698541	24	0.0%	0	
Case8- heart1	GSM4698542	78	0.0%	0	
Case8-lung1 RLL	GSM4698544	3,150	5.0%	0	
Case8-lung2 RUL	GSM4698545	200	51.0%	0	
Case8-lung3 LLL	GSM4698546	24,527	1.8%	46	0.0%
Case8-lung4 RML	GSM4698547	5,820	2.1%	0	
Case8-lung5 LUL	GSM4698548	102	0.0%	0	
Case9- lung1RLL	GSM4698549	45,539	6.4%	56	5.4%
Case9- lung2 RML	GSM4698550	154,157	9.6%	405	42.5%
Case9- lung3RUL	GSM4698551	138,578	8.1%	173	3.5%
Case9-lung4 LUL	GSM4698552	361,535	4.8%	652	5.8%
Case9- lung5LLL	GSM4698553	179,729	14.5%	145	35.2%
Case10- lung2 LLL	GSM4698522	112	0.0%	0	
Case10- lung3 RLL	GSM4698523	22	0.0%	0	

Case11 -bowel1	GSM4698524	92	0.0%	0	
Case11-lung1RML	GSM4698526	72,029	3.2%	141	2.8%
Case11-lung32RUL	GSM4698527	17,256	12.7%	13	0.0%
Case11-lung3RLL	GSM4698528	1,328	3.5%	0	
CaseA-lung	GSM4698554	1,202	4.0%	0	
CaseB-lung	GSM4698555	6	33.3%	0	
CaseC-lung	GSM4698556	168,935	1.5%	47	0.0%
CaseD-lung	GSM4698557	43,750	6.8%	9	0.0%

Table S3. Summary of negative strand viral and human-viral chimeric RNA-seq reads from BALF cells of COVID-19 patients (bulk analysis of published single-cell RNA-seq data, Liao et al., GSE145926)

Patient	GEO accession number	CoV2 reads	Negative strand CoV2 read fraction	Human-CoV2 chimeric reads	Negative strand CoV2 (in chimeric RNAs) read fraction
C143 (severe)	GSM4339771	1,525	18.6%	1	0.0%
C145 (severe)	GSM4339773	34,439	12.7%	16	0.0%
C146 (severe)	GSM4339774	893,327	15.3%	223	1.4%
C148 (severe)	GSM4475051	1,251	22.0%	0	
C149 (severe)	GSM4475052	89,928	19.6%	23	0.0%
C152 (severe)	GSM4475053	32,665	18.5%	5	0.0%

Table S4. PCR primers used in this study

Name	Sequences
N1	Forward: GACCCCAAAATCAGCGAAAT<br>Reverse: TCTGGTTACTGCCAGTTGAATCTG
N2	Forward: GGGAGCCTTGAATACACCAAAA<br>Reverse: TGTAGCACGATTGCAGCATTG
N3	Forward: GGGGAACTTCTCCTGCTAGAAT<br>Reverse: CAGACATTTTGCTCTCAAGCTG
N4	Forward: AAATTTTGGGGACCAGGAAC<br>Reverse: TGGCACCTGTGTAGGTCAAC
N (for cloning complete NC gene)	Forward: ATGTCTGATAATGGACCCCAAAAT<br>Reverse: TTAGGCCTGAGTTGAGTCAGC
HSPA1A	Forward: ATCTCCACCTTGCCGTGTT<br>Reverse: ATCCAGTGTTCCGTTTCCAG

Dataset S1 (separate file). Sanger sequencing results of the complete SARS-CoV-2 NC gene cloned from large-fragment cell genomic DNA from LINE1-overexpressing, SARS-CoV-2-infected HEK293T cells.

Dataset S2 (separate file). Summary of chimeric read sequences from Nanopore sequencing of DNA from LINE1-overexpressing, SARS-CoV-2-infected HEK293T cells.

Dataset S3 (separate file). Summary of chimeric sequences from Illumina paired-end whole genome sequencing of DNA from LINE1-overexpressing, SARS-CoV-2-infected HEK293T cells.

Dataset S4 (separate file). Summary of chimeric sequences from Tn5 tagmentation-mediated DNA integration site enrichment sequencing of DNA from SARS-CoV-2 infected HEK293T or Calu3 cells.

Update for Zhang et al., Reverse-transcribed SARS-CoV-2 RNA can integrate into the genome of cultured human cells and can be expressed in patient-derived tissues.

In our publication[1], we reported a total of 63 integrations of SARS-CoV-2 sequence in the genome of HEK293T cells overexpressing LINE1, by Nanopore whole-genome sequencing (Table 1). We recovered 2 integrations with human sequences flanking a viral sequence on both sides (Fig. 1B-D, S2A-C), showing a 20-bp or 13-bp target-site duplication as strong evidence for LINE1 mediated integration[2,3]. For the other 61 integrations we recovered only one-side flanking the human sequence. As we reported, ~67% of all 63 integrations included a LINE1 endonuclease recognition sequence[4] at the flanking human sequence that were either directly linked to the 3' end (poly-A tail) of the viral sub-genomic RNA sequence, or within a potential target site duplication (8-27 bp) that were linked to the 5' end of viral sequences, as evidence for LINE1 mediated integration. Another prediction of LINE1-mediated retrointegration is the presence of a poly-A tract at the 3' end of integrant[5-9]. As summarized in the table we found "poly-A tracts" ranging from 2 – 65 bp for these integrations at the junctions of the LINE1 endonuclease recognition sequence (e.g. 5'-TTTT|A-3') and the shared 3' end of the integrated viral sub-genomic RNA sequence (lower strand: 5'-polyT-GTCATTCTC…-3'). This supports a LINE1 mediated integration mechanism. The figure details the sequences of all integrants.

Table S5. Summary of Nanopore chimeric sequences with 3'-end of SARS-CoV-2 sub-genomic RNA sequence directly linked to a LINE1 endonuclease recognition sequence in the human genome

	Chromosome	L1 endonuclease recognition sequence[4]	"poly-A tract" length (bp)*	Genomic feature	Illustrated in figures[1]
1	chr1	**CTTT|A**	7	intron (GALNT2)	Fig. S2D-F
2	chr1	**CTTT|C**	31	intron (PEX14)	
3	chr1	**TCTT|A**	21	intron (AL359924.1, non-coding RNA)	
4	chr1	TTTTTTC**TTTT|A**	40	exon (ZNF644)	
5	chr1	TTTAT**TTTT|A**	21	intron (KIAA0319L)	
6	chr2	TTTT**ATTT|A**	3	intron (EML4-AS1, non-coding RNA)	
7	chr3	TT**TCTT|C**(?)	16	intron (AC012020.1, non-coding RNA)	
8	chr4	TT**TTCT|G**	35	intron (AC097528.1, non-coding RNA)	
9	chr5	**TTTT|A**	2	intron (CCDC127)	
10	chr6	**TTCT|A**	4	intergenic	
11	chr6	T**TTTC|A**	7	intergenic	
12	chr7	T**TTTT|C**	8	intron (NRCAM)	
13	chr7	**AGTT|C**	2	intergenic	
14	chr9	**TTTT|C**	19	intergenic	
15	chr9	T**TGTT|A**	4	exon (3'UTR) (SCAI)	
16	chr10	TTT**TTTT|C**	16	intergenic	
17	chr11	T**TTTC|A**	8	exon (NEAT1, non-coding RNA)	
18	chr11	TT**TCTT|A**	51	intron (TRIM44)	
19	chr12	TT**TTTC|A**	22	intron (AC048352.1, LINC02378, non-coding RNAs)	
20	chr12	**TTTT|C**	65	intergenic	

<table>
<tr><td></td><td></td><td></td><td></td><td></td><td></td></tr>
<tr><td>21</td><td>chr12</td><td>TTTTT|A</td><td>3</td><td>intergenic</td><td></td></tr>
<tr><td>22</td><td>chr12</td><td>TTTTCTT|A</td><td>4</td><td>intron (POLR3B)</td><td></td></tr>
<tr><td>23</td><td>chr13</td><td>TTCT|A</td><td>13</td><td>intergenic</td><td></td></tr>
<tr><td>24</td><td>chr13</td><td>TCTT|A</td><td>5</td><td>intergenic</td><td></td></tr>
<tr><td>25</td><td>chr14</td><td>TTTTTTTTTTTTTTTT|G</td><td>18</td><td>intergenic</td><td></td></tr>
<tr><td>26</td><td>chr16</td><td>TTTTT|A</td><td>10</td><td>intron (CDR2)</td><td></td></tr>
<tr><td>27</td><td>chr18</td><td>TTTTCTT|G</td><td>21</td><td>intron (LINC01915, non-coding RNA)</td><td></td></tr>
<tr><td>28</td><td>chr22</td><td>TTCTT|A</td><td>10</td><td>intron (IGLV10-54)</td><td>Fig. S2A-C</td></tr>
<tr><td>29</td><td>ChrX</td><td>TTTTT|A</td><td>33</td><td>intron (DMD)</td><td></td></tr>
<tr><td>30</td><td>ChrX</td><td>TTTTTT|C</td><td>36</td><td>intergenic</td><td></td></tr>
<tr><td>31</td><td>ChrX</td><td>TTCT|A</td><td>4</td><td>intergenic</td><td>Fig. 1B-D</td></tr>
<tr><td>32</td><td>ChrX</td><td>TTTTCTT|C(?)</td><td>61</td><td>exon (SLITRK2)</td><td></td></tr>
</table>

*The “poly-T” of the human sequence at the junction is counted. This is the site where the viral RNA “poly-A” sequence annealed for “target-primed reverse transcription”.

References

1 Zhang, L. *et al.* Reverse-transcribed SARS-CoV-2 RNA can integrate into the genome of cultured human cells and can be expressed in patient-derived tissues. *Proceedings of the National Academy of Sciences* **118**, doi:10.1073/pnas.2105968118 (2021).

2 Kazazian, H. H., Jr. & Moran, J. V. Mobile DNA in Health and Disease. *N Engl J Med* **377**, 361-370, doi:10.1056/NEJMra1510092 (2017).

3 Kaessmann, H., Vinckenbosch, N. & Long, M. RNA-based gene duplication: mechanistic and evolutionary insights. *Nature reviews. Genetics* **10**, 19-31, doi:10.1038/nrg2487 (2009).

4 Morrish, T. A. *et al.* DNA repair mediated by endonuclease-independent LINE-1 retrotransposition. *Nature genetics* **31**, 159-165, doi:10.1038/ng898 (2002).

5 Moran, J. V. *et al.* High Frequency Retrotransposition in Cultured Mammalian Cells. *Cell* **87**, 917-927, doi:10.1016/S0092-8674(00)81998-4 (1996).

6 Esnault, C., Maestre, J. & Heidmann, T. Human LINE retrotransposons generate processed pseudogenes. *Nature genetics* **24**, 363-367, doi:10.1038/74184 (2000).

7 Dewannieux, M., Esnault, C. & Heidmann, T. LINE-mediated retrotransposition of marked Alu sequences. *Nature genetics* **35**, 41-48, doi:10.1038/ng1223 (2003).

8 Garcia-Perez, J. L., Doucet, A. J., Bucheton, A., Moran, J. V. & Gilbert, N. Distinct mechanisms for trans-mediated mobilization of cellular RNAs by the LINE-1 reverse transcriptase. *Genome research* **17**, 602-611, doi:10.1101/gr.5870107 (2007).

9 Hancks, D. C., Ewing, A. D., Chen, J. E., Tokunaga, K. & Kazazian, H. H., Jr. Exon-trapping mediated by the human retrotransposon SVA. *Genome research* **19**, 1983-1991, doi:10.1101/gr.093153.109 (2009).

Chr1:

Read: 07573090-2aaf-49c6-8a1d-d094d8a66847

Human: ...CTCGGCCTCCTGCTTTTCTCTTTA...
Chi read: 5'...CTCGGCCTCCTGCTTTTCTCTTTTTTTGTCATTCTCCT...3'
CoV2: TTTTTTTGTCATTCTCCT...

LINE1 endonuclease recognition sequence: CTTT|A
"PolyA tract" length: 7 bp
Integration site: intron (GALNT2)

Read: e05cbd8d-36b5-4dc2-b54b-f5ef36ca35a0

Human: ...CAGTGGCGCTGGCTTCTGCCTTTC...
Chi read: 5'...CAGTGGCGCTGGCTTCTGCCTTTTTTTTTTTTTTTTTTTTTTTTTTTTTTTGTCATTCTCCT...3'
CoV2: TTTTTTTTTTTTTTTTTTTTTTTTTTTTTTTGTCATTCTCCT...

LINE1 endonuclease recognition sequence: CTTT|C
"PolyA tract" length: 31 bp
Integration site: intron (PEX14)

Read: be93c20e-0591-4e2b-a633-df92b6a36e04

Human: ...CAAATGAACAAATACATCTTA...
Chi read: 5'... CAAATGAACAAATACATCTTGATTTTTTTTTTTTTTTTTTGTCATTCTC...3'
CoV2: T TT TTTTTTTTTTTTTTTTGTCATTCTC...

LINE1 endonuclease recognition sequence: TCTT|A
"PolyA tract" length: 21 bp
Integration site: intron (AL359924.1, non-coding RNA)

Read: 80097bd4-2c11-40f8-9238-59826c54606b

Human: ...AAGGATGTCATTTTTTCTTTTA...
Chi read: 5'...AAGGATGTCATTTTTTCTTTTTTTTTTTTTTTTTTTTTTTTTTTTTTTTTTTTTTTTTTGTCATTCTCCT...3'
CoV2: TTTTTTTTTTTTTTTTTTTTTTTTTTTTTTTTTTTTTTTTGTCATTCTCCT...

LINE1 endonuclease recognition sequence: TTTTTTCTTTT|A
"PolyA tract" length: 40 bp
Integration site: exon (ZNF644)

Read: f40cad59-4beb-49a0-87c5-7642024de8e7

Human: ...AACAAAGACCAATTTATTTTTA...
Chi read: 5'...AACAAAGACCAATTTATTTTTTTTTGTTTTTTTTTTTTGTCATTCTCCTA...
CoV2: TTTTTTTTT TTTTTTTTTTTTGTCATTCTCCTA...

LINE1 endonuclease recognition sequence: TTTATTTTT|A
"PolyA tract" length: 21 bp
Integration site: intron (KIAA0319L)

Chr2:

Read: 40d42d5b-7125-4200-8d88-0ea079267ab0

Human: ...TCATGTCAGAAGAACTGCTTTTATTTA...
Chi read: 5'...TCATGTCAGAAGAACTGC TTTATTTGTCATTCTCCTAAGA...3'
CoV2: TTTGTCATTCTCCTAAGA...

LINE1 endonuclease recognition sequence: TTTTATTT|A
"PolyA tract" length: 3 bp
Integration site: intron (EML4-AS1, non-coding RNA)

Chr3:

Read: d9ff2a50-f4fb-482c-ba21-6a54d47c7bf1

Human: ...TTCAGGATCACCTTTCTTC...
Chi read: 5'...TTCAGGATCACCTTTCTTTTTTTTTTTTTGTCATTCTCCT...3'
CoV2: TTT TTTTTTTTTTTTTGTCATTCTCCT...

LINE1 endonuclease recognition sequence: TTTCTT|C
"PolyA tract" length: 16 bp
Integration site: intron (AC012020.1, non-coding RNA)

Chr4:

Read: 28ea733a-fb5f-484a-8f69-c60135693069

Human: ...AGAGACTTTTCTG...
Chi read: 5'...AGAGACTTTTCTGTTTTTTTTTTTTTTTTTTTTTTTTTTTTTTGTCATTCTC...3'
CoV2: TTTT T TTTTTTTTTTTTTTTTTTTTTTTTTTTTTGTCATTCTC...

LINE1 endonuclease recognition sequence: TTTTCT|G
"PolyA tract" length: 35 bp
Integration site: intron (AC097528.1, non-coding RNA)

Chr5:

Read: da820d14-8fdd-400b-b319-751055955437

```
Human:      …TAGTCAGAGTAATTTTA…
Chi read:  5'…TAGTCAGAGTAA   TTGTCATTCT  CTAAGAAGCTATT…3'
CoV2:                         TTGTCATTCTCCTAAGAAGCTATT…
```

LINE1 endonuclease recognition sequence: TTTT|A
"PolyA tract" length: 2 bp
Integration site: intron (CCDC127)

Chr6:

Read: 5b2b7b8d-79ec-4378-a055-246618f76bb4

```
Human:      …TGGAAGTATTATTCTA…
Chi read:  5'…TGGAAGTATTATTCTTGTCATTCTCCTAAG…3'
CoV2:                    TT  TTGTCATTCTCCTAAG…
```

LINE1 endonuclease recognition sequence: TTCT|A
"PolyA tract" length: 4 bp
Integration site: intergenic

Read: fb8bd42f-a1d6-4e52-99ff-f2c0d021a59f

```
Human:      …GGATGCTTCAGTTATTTTCA…
Chi read:  5'…GGATGCTTCAGTTATTTTCTTTGTCATTCTCCTAAG…3'
CoV2:                         TTTT   TTTGTCATTCTCCTAAG…
```

LINE1 endonuclease recognition sequence: TTTTC|A
"PolyA tract" length: 7 bp
Integration site: intergenic

Chr7:

Read: d296e986-48f2-4d72-93e6-b53456f8e1c4

Human: ...ATGCATTGATTTTTTGAACAGTTTTTC...
Chi read: 5'...ATGCATTGAT TTTTGAACAGTTTTTTTTGTCATTTCTCCTAAGAAG...3'
CoV2: TTTTTTTTGTCATT CTCCTAAGAAG...

LINE1 endonuclease recognition sequence: TTTTT|C
"PolyA tract" length: 8 bp
Integration site: intron (NRCAM)

Read: 9bf30fba-3195-4241-b2e3-e5ab595e6e1d

Human: ...CACCAGTGGAGTTC...
Chi read: 5'...CACCAGTGGAGTTGTCATTTTCTCCTAAGAAGCTATTAA...3'
CoV2: TTGTCA TTCTCCTA GAAGCTATTAA...

LINE1 endonuclease recognition sequence: AGTT|C
"PolyA tract" length: 2 bp
Integration site: intergenic

Chr9:

Read: 7a893df8-8b8f-47fd-9286-b7d4e7ebee4e

Human: ...CATAGAACTGGGTGGCTTAATTTTC...
Chi read: 5'...CATAGAACTGGGTGGCTTAATTTTTTTTTTTTTTTTTTTGTCATTCTC TAAGAAGCTATT...3'
CoV2: TTTTTTTTTTTTTTTTTTTGTCATTCTCCTAAGAAGCTATT...

LINE1 endonuclease recognition sequence: TTTT|C
"PolyA tract" length: 19 bp
Integration site: intergenic

Read: 6a43b132-635c-4c9b-9e29-04790f6770b1

Human: ...CAGACAAGCACAATTGTTA...
Chi read: 5'...CAGACAAGCACAATTGTTGTCATTCTCCTA...3'
CoV2: TT TTGTCATTCTCCTA...

LINE1 endonuclease recognition sequence: TTGTT|A
"PolyA tract" length: 4 bp
Integration site: exon (3'UTR) (SCAI)

Chr10:

Read: 8975c59e-bc5e-4d71-ae28-55d1819e8032

```
Human:      …TACTTTCTTGCATATGATTTTTTTC…
Chi read:  5'…TACTTTCT  GCATATGATTTTTCTTTTTTTTTTTGTCATTCTCC…3'
CoV2:                          TTTTT  TTTTTTTTTTTGTCATTCTCC..
```

LINE1 endonuclease recognition sequence: TTTTTTT|C
"PolyA tract" length: 16 bp
Integration site: intergenic

Chr11:

Read: b365ab7a-6514-459b-bd3c-a4689f41d9ab

```
Human:      …CAATTTCACTTCGCTCATGATTTTCA…
Chi read:  5'…CAATTTCACTTCGCTCATGATTTTCTTTTGTCATTCTCCTAAG…3'
CoV2:                                TTTT  TTTTGTCATTCTCCTAAG…
```

LINE1 endonuclease recognition sequence: TTTTC|A
"PolyA tract" length: 8 bp
Integration site: exon (NEAT1, non-coding RNA)

Read: c90e0881-3da9-4730-be43-227d9a2cbb05

```
Human:      …AGAGTGTAGTGTTTCTTA…
Chi read:  5'…AGAGTGTAGTGTTTCTTTTTTTTTTTTTTTTTTTTTTTTTTTTTTTTTTTTTTTTTTTTTTTTTGTCATTCTCCT…3'
CoV2:                       TTT  TTTTTTTTTTTTTTTTTTTTTTTTTTTTTTTTTTTTTTTTTTTTTTTTGTCATTCTCCT…
```

LINE1 endonuclease recognition sequence: TTTCTT|A
"PolyA tract" length: 51 bp
Integration site: intron (TRIM44)

Chr12:

Read: 17c45e22-69ae-4f6e-93b4-31d3a81d6f9b

```
Human:      …GAATTTCCAGGCTGTTTTTCA…
Chi read:  5'…GAATTTCCAGGCTG  TTTTCTTTTTTTTTTTTTTTTTTGTCACATTCTCCTAAGAAGCTAT…3'
CoV2:                           TTTT  TTTTTTTTTTTTTTTTTTGT    CATTCTCCTAAGAAGCTAT…
```

LINE1 endonuclease recognition sequence: TTTTTC|A

“PolyA tract” length: 22 bp
Integration site: intron (AC048352.1, LINC02378, non-coding RNAs)

Read: 69963961-b72e-4193-8fd9-29350e837663

Human: …ACCTTCCATTGTCTTTTC…
Chi read: 5’…ACCTTCTATTGTCTTTTTTCTTTTTTTTTT(Tx30)TTTTTTTTTTTTTTTTTTTGTCATTCTCCTAAGAA…3’
CoV2: TTTTTT TTTTTTTTTT(Tx30)TTTTTTTTTTTTTTTTTTTGTCATTCTCCTAAGAA…

LINE1 endonuclease recognition sequence: TTTT|C
“PolyA tract” length: 65 bp
Integration site: intergenic

Read: 0e6049f7-fdd2-49bd-b35b-131ed9791e88

Human: …GCCTTTTCTGTTTCCTTTAAATTTTTA…
Chi read: 5’…GCCTTTTCTGTTTCCTTTAAA TTTGTCATTCTCCTAAGAAGCT…3’
CoV2: TTTGTCATTCTCCTAAGAAGCT…

LINE1 endonuclease recognition sequence: TTTTT|A
“PolyA tract” length: 3 bp
Integration site: intergenic

Read: c9e4ccb2-7edc-4535-af71-504f0670cf04

Human: …TGTGCCACATTTTCTTA…
Chi read: 5’…TGTGCCACATCTTCTTGTCAATTCTCCTAAGAAGCTATTA…3’
CoV2: TT TTGTCA TTCTCCTAAGAAGCTATTA…

LINE1 endonuclease recognition sequence: TTTTCTT|A
“PolyA tract” length: 4 bp
Integration site: intron (POLR3B)

Chr13:

Read: 94f90063-f0d8-4a56-9f78-cea4b239b5fa

Human: …ACATTAGAGTTTTCTTCTA…
Chi read: 5’…ACATTAGAGTTTTCTTCTATTTAAGTTTTTTTGTCATTCTCCTAAGAAGC…3’
CoV2: TT T TTT TTTTTTTGTCATTCTCCTAAGAAGC…

LINE1 endonuclease recognition sequence: TTCT|A
"PolyA tract" length: 13 bp
Integration site: intergenic

Read: 6e548530-47d4-4b2f-8665-b2107fc66d32

Human: ...GGTATTTCCTAGGTTATCTTA...
Chi read: 5'...GGTATTTCCTAGGTTATCTTATTGTCATTCTCCT...3'
CoV2: T TT TTGTCATTCTCCT...

LINE1 endonuclease recognition sequence: TCTT|A
"PolyA tract" length: 5 bp
Integration site: intergenic

Chr14:

Read: 50eecf64-c7a5-4966-a519-3e5c611cc423

Human: ...CACATTGTTTGCTTTTTTTTTTTTTTTTTG...
Chi read: 5'...CACATTGTTTGCTTTTTTTTTTTTTTTTTTGTC TACTCCTA GAAGC GTAAAAATCACATGGGGATAG...3'
CoV2: TTTTTTTTTTTTTTTTTTGTCATTCTCCTAAGAAGCTATTAAAATCACATGGGGATAG...

LINE1 endonuclease recognition sequence: TTTTTTTTTTTTTTTTT|G
"PolyA tract" length: 18 bp
Integration site: intergenic

Chr16:

Read: 0c381657-f571-4cdc-b8de-17bc6a224ade

Human: ...AAAGCAACTTTTTA...
Chi read: 5'...AAAGCAACTTTTTTTTTTGTCATTCTCCTA...3'
CoV2: TTTTTTTTTTGTCATTCTCCTA...

LINE1 endonuclease recognition sequence: TTTTT|A
"PolyA tract" length: 10 bp
Integration site: intron (CDR2)

Chr18:

Read: 028774db-8163-4dde-b7af-16c7d7b7cde2

Human: ...TCAACTTCAATTTATTTTTTAAAATTTTCTTG ...
Chi read: 5'...TCAACTTCAATTTATTTTTTAAAATTTTCTTCCTTTTTTTTTTTTTTTGTCATTCTCCTAAGAAGCTATT...3'
CoV2:   TTTT TT   TTTTTTTTTTTTTTTGTCATTCTCCTAAGAAGCTATT...

LINE1 endonuclease recognition sequence: TTTTCTT|G
"PolyA tract" length: 21 bp
Integration site: intron (LINC01915, non-coding RNA)

Chr22:

Read: f40cad59-4beb-49a0-87c5-7642024de8e7

Human: ...AGGTTTGGTGACATCACTTCTTA...
Chi read: 5'...AGGTTTGGTGACATCACTTCTTTTTTTTGTCATTCTC TAAGAAGCTATT...3'
CoV2:   TT TTTTTTTTGTCATTCTCCTAAGAAGCTATT...

LINE1 endonuclease recognition sequence: TTCTT|A
"PolyA tract" length: 10 bp
Integration site: intron (IGLV10-54)

ChrX:

Read: 1233b662-c642-472b-9529-73286cc3f473

Human: ...TTAGGAATGCATATTCTGATTTTTA...
Chi read: 5'...TTAGGAATGCATATTCTGATTTTTTTTTTTTTTTTTTTTTTTTTTTTTTTTTTGTCATTCTCCTAAGAAG...3'
CoV2:   TTTTTTTTTTTTTTTTTTTTTTTTTTTTTTTTTGTCATTCTCCTAAGAAG...

LINE1 endonuclease recognition sequence: TTTTT|A
"PolyA tract" length: 33 bp
Integration site: intron (DMD)

Read: 502e6323-b284-442e-a039-c28e14a92c06

Human: ...GACTTTTTGCACCTTTTTTC...
Chi read: 5'...GACTTTTTGCACCTTTTTTTTTTTTTTTTTTTTTTTTTTTTTTTTTTTTTGTCATTCTCCTAAGAAG...3'
CoV2:   TTTTTTTTTTTTTTTTTTTTTTTTTTTTTTTTTTTTGTCATTCTCCTAAGAAG...

LINE1 endonuclease recognition sequence: TTTTTT|C
"PolyA tract" length: 36 bp
Integration site: intergenic

Read: b1c4ffb8-480b-4a10-9461-6f1393119003

Human: ...TCAATTGCTATTGCTTCTA...
Chi read: 5'...TCAATTGCTATTGCTTCTTGTCATTCTC TAAGAA...3'
CoV2: TT TTGTCATTCTCCTAAGAA...

LINE1 endonuclease recognition sequence: TTCT|A
"PolyA tract" length: 4 bp
Integration site: intergenic

Read: 4757692e-e0cd-46b5-b3b2-163dc1d6a2ad

Human: ...TATTCAGTACGTTTTCCTTTTCTTC...
Chi read: 5'...TATTCAGTACGTTTTCCTTTTCTTTTTTTTTTTTT(Tx25)TTTTTTTTTTTTTTTTTTTGTCATTCTCCTAA...3'
CoV2: TTTT TTTTTTTTTTTTT(Tx25)TTTTTTTTTTTTTTTTTTTGTCATTCTCCTAA...

LINE1 endonuclease recognition sequence: TTTTCTT|C
"PolyA tract" length: 61 bp
Integration site: exon (SLITRK2)
